# Differential responses of soil microbial metabolic limitation and carbon use efficiency to long-term grazing exclusion across three grassland types in Xinjiang

**DOI:** 10.3389/fmicb.2026.1905160

**Published:** 2026-09-11

**Authors:** Asitaiken Julihaiti, Sun Zongjiu, Guo Jinhua, Jing Yisheng, Dong Yiqiang

**Affiliations:** 1 School of Grassland, Xinjiang Agricultural University, Ürümqi, China; 2 Key Laboratory of Grassland Resources and Ecology Autonomous Region, Xinjiang Agricultural University, Xinjiang, Ürümqi, China; 3 Key Laboratory of Grassland Resources and Ecology, Ministry of Education, Western Arid Region, Ürümqi, China

**Keywords:** carbon use efficiency, ecoenzymatic stoichiometry, grassland type, grazing exclusion, microbial nutrient limitation

## Abstract

**Introduction:**

Long-term grazing exclusion is widely used to restore degraded grasslands, yet whether its effects on soil microbial metabolism–particularly nutrient limitation and carbon use efficiency (CUE)–vary among grassland types remains poorly understood.

**Methods:**

We investigated three typical grassland types in Xinjiang, China: temperate desert (TD), temperate steppe (TS), and mountain meadow (MM), under 12 years of grazing exclusion and free grazing. We measured soil extracellular enzyme activities, assessed microbial nutrient limitation via vector analysis, and calculated microbial CUE.

**Results:**

Grazing exclusion produced contrasting effects among grassland types. In TD, all three enzyme activities significantly increased (*P* < 0.01); in TS, C- and N-acquiring enzymes decreased (*P* < 0.05); and in MM, only C-acquiring enzyme increased (*P* < 0.001), accompanied by a significant reduction in CUE (*P* < 0.01). Grazing exclusion generally intensified microbial C limitation (increased vector length) while alleviating N limitation (vector angles shifted toward 45°), though all vector angles remained below 45°, indicating persistent N limitation. Partial least squares structural equation modeling revealed distinct regulatory pathway: an indirect “soil → dissolved nutrients → microbial biomass” pathway in TD; a direct “soil → microbial biomass” pathway in TS; and a longer cascade pathway in MM that enhanced C limitation but reduced CUE.

**Discussion:**

Our findings demonstrate that grazing exclusion effects on microbial metabolism are strongly grassland-type dependent. Grazing exclusion promoted favorable microbial functional responses in TD and TS, but intensified C limitation and reduced CUE in MM. Vector length and angle serve as sensitive indicators of microbial nutrient limitation during restoration. These findings highlight the need for ecosystem-specific restoration strategies in arid and semi-arid regions.

## 1 Introduction

Grasslands are among the largest terrestrial ecosystems on Earth, covering approximately 25% of the land surface and playing a vital role in ecosystem services such as carbon sequestration and biodiversity maintenance (Hewins et al., 2015; Eldridge and Delgado-Baquerizo, 2017). In China, grasslands account for about 30% of the national land area and serve as an important carbon sink (Yang et al., 2022). However, overgrazing and other anthropogenic activities have led to varying degrees of degradation in more than 90% of China's natural grasslands, manifested as declines in vegetation cover, productivity, and soil fertility (Gang et al., 2014). Grassland degradation substantially impairs carbon uptake capacity and ecosystem services (Yang et al., 2022; Bardgett et al., 2021), and even after the removal of grazing, the recovery of original dominant species usually takes more than 20 years (Lü et al., 2025). Therefore, developing effective restoration measures for degraded grasslands is of great importance for sustaining livestock husbandry and enhancing carbon sink capacity.

Soil microorganisms are key drivers of terrestrial biogeochemical cycles; they regulate the decomposition of soil organic matter and the transformation of nutrients by secreting extracellular enzymes (Bardgett and Van Der Putten, 2014; Li S. Y. et al., 2024). Microbial metabolism essentially represents an equilibrium between nutrient demand and environmental nutrient availability (Moorhead et al., 2013). Soil extracellular enzyme activity (EEA) is a sensitive indicator of microbial resource allocation during energy and nutrient (C, N, P) acquisition (Fanin et al., 2016). Based on ecological stoichiometry theory (EST), soil extracellular enzyme stoichiometry (EES) can be quantified using EEA ratios to predict nutrient availability and microbial metabolic limitation (Li et al., 2019). Commonly measured extracellular enzymes include β-1,4-glucosidase (BG, C-acquisition), β-1,4-N-acetylglucosaminidase (NAG) and leucine aminopeptidase (LAP, N-acquisition), and alkaline phosphatase (AP, P-acquisition) (Sinsabaugh et al., 2008). The activities of these enzymes can serve as effective proxies for microbial resource allocation to C, N, and P (Deng et al., 2019; Fanin et al., 2016).

To more intuitively assess microbial metabolic limitations, Moorhead et al. (2016) proposed a vector analysis model that quantifies the relative investments of microbes in C vs. nutrients and P vs. N by calculating vector length and vector angle. Vector length reflects the degree of C limitation, whereas vector angle indicates N limitation (< 45°) or P limitation (>45°). This method has been widely applied in studies of grassland degradation, vegetation restoration, and land-use change, demonstrating high sensitivity to microbial nutrient limitation (Dong et al., 2019; Yang et al., 2020; Wu et al., 2025). Furthermore, microbial carbon use efficiency (CUE)-the proportion of absorbed carbon allocated to biomass synthesis-is a key indicator for predicting soil carbon dynamics and sequestration potential (Feng et al., 2022; Manzoni et al., 2012). High CUE promotes microbial necromass accumulation and carbon sequestration, whereas low CUE implies that more carbon is respired, thereby increasing soil organic carbon mineralization (Xu Q. C. et al., 2024). CUE is jointly regulated by multiple factors, including microbial community composition, soil nutrient status, and plant litter quality (Malik et al., 2019), and is closely related to soil C:N:P stoichiometry; stoichiometric imbalance constrains microbial C and N use efficiencies (Liao et al., 2024). Therefore, integrating vector analysis with CUE measurements can help elucidate the microbial mechanisms underlying soil carbon pool dynamics.

Grazing exclusion (fencing) is a nature-based restoration measure widely used in degraded grasslands worldwide (Cheng et al., 2016). By eliminating livestock foraging and trampling, grazing exclusion promotes plant biomass, soil nutrient accumulation, and microbial diversity (Wang et al., 2020). However, the effects of grazing exclusion on soil EEA, EES, and microbial CUE are still poorly understood. Previous studies have shown that grassland management practices alter soil resource stoichiometry, leading to stoichiometric imbalances between soil and microorganisms, thereby affecting microbial nutrient limitation (Wang et al., 2020; Zhong et al., 2020; Zhang et al., 2024). In the desert steppe of Inner Mongolia, long-term grazing aggravated microbial C and P limitations, whereas moderate grazing alleviated C limitation (Li H. Y. et al., 2024). Yet, little is known about how grazing exclusion regulates microbial nutrient limitation and CUE across different grassland types (e.g., temperate desert, temperate steppe, and mountain meadow), and the underlying environmental drivers. Notably, due to differences in climate, vegetation, and soil background, the effects of grazing exclusion may show divergent regulation pathways among grassland types. However, comparative studies across multiple grassland types remain scarce, and few have integrated enzyme activities, vector characteristics, CUE, and structural equation modeling to dissect the regulatory mechanisms of grazing exclusion.

To address these knowledge gaps, this study focused on three typical grassland types in Xinjiang-temperate desert (TD), temperate steppe (TS), and mountain meadow (MM)-and compared 12-year grazing exclusion with free grazing. The objectives were to: (1) elucidate the differential effects of long-term grazing exclusion on soil extracellular enzyme activities, microbial nutrient limitation (vector characteristics), and microbial CUE across the three grassland types; and (2) identify the key driving pathways through which grazing exclusion regulates microbial nutrient limitation and CUE by integrating vegetation, soil physicochemical properties, dissolved nutrients, and microbial biomass. We hypothesized that: (1) long-term grazing exclusion would increase soil resource availability by enhancing vegetation recovery and soil nutrient accumulation, thereby altering soil C, N, and P stoichiometric balance; (2) changes in soil resource availability and stoichiometric balance would regulate microbial nutrient limitation and CUE, as reflected by variations in microbial vector characteristics and metabolic allocation strategies; and (3) the magnitude and direction of these responses would differ among contrasting grassland ecosystems due to differences in environmental conditions and plant–soil–microbe interactions. The results of this study will provide a microbiological basis for the restoration of different types of degraded grasslands via grazing exclusion and support the development of differentiated management strategies at the regional scale.

## 2 Materials and methods

### 2.1 Study area and experimental design

The study area is situated on the eastern section of the northern slope of the Tianshan Mountains in Xinjiang, covering Fukang City, Mulei County, and Qitai County within the Changji Hui Autonomous Prefecture, where the basic characteristics of sampling sites are summarized in Table 1. Specifically, the Fukang area is characterized by temperate desert with sierozem soil of sandy texture, dominated by *Haloxylon ammodendron* and *Seriphidium santolinum* and associated with *Salsola collina*; in contrast, the Mulei area features temperate steppe with chernozem or chestnut soil of loam texture, mainly dominated by *Stipa capillata, Festuca ovina*, and *Carex liparocarpos* and associated with *Sibbaldianthe bifurca*; furthermore, the Qitai area is identified as mountain meadow with chernozem soil of loam texture, primarily dominated by *Achillea millefolium, Geranium carolinianum*, and *Thalictrum aquilegiifolium* and associated with species such as *Codonopsis pilosula, Leontopodium leontopodioides*, and *Campanula glomerata*.

**Table 1:** Plant community and soil physicochemical properties under grazing and grazing exclusion across three grassland types (temperate desert, temperate steppe, mountain meadow).

Parameters		Temperate desert_FG_	Temperate desert_GE_	Temperate steppe_FG_	Temperate steppe_GE_	Mountain meadow_FG_	Mountain meadow_GE_
Site characteristics	Latitude and longitude	44° 23′ 9′′ N, 88° 8′ 48′′ E	44° 23′ 9′′ N, 88° 8′ 48′′ E	43° 40′ 34′ N, 90° 14′ 58′′ E	43° 40′ 34′′ N, 90° 14′ 58′ E	43° 37′ 3′ N, 89° 35′ 45′′ E	43° 37′ 3′′ N, 89° 35′ 45′′ E
	Altitude (m)	514.0	514.0	1987	1987	2093	2093
	Mean annual precipitation (mm)	145.0	145.0	296.6	296.6	420	420
	Mean annual temperature (°C)	5.9	5.9	6.0	6.0	5.5	5.5
Plant characteristics	Aboveground biomass (g·m^−2^)	16.91 ± 3.20	12.31 ± 2.28	45.36 ± 4.38	109.07 ± 7.49	101.25 ± 5.56	179.01 ± 17.21
	Belowground biomass (g·m^−2^)	405 ± 6.52	303 ± 19.21	2048 ± 45.13	2497 ± 54.81	1173 ± 63.32	2153 ± 51.61
	Shannon–Wiener index	0.5648 ± 0.09	0.5048 ± 0.08	1.66 ± 0.08	2.17 ± 0.05	1.89 ± 0.05	2.22 ± 0.09
Soil properties	pH	9.234 ± 0.08	9.384 ± 0.08	7.332 ± 0.07	6.952 ± 0.05	7.132 ± 0.07	7.25 ± 0.03
	Soil water content (%)	0.03 ± 0.01	0.062 ± 0.01	0.222 ± 0.01	0.306 ± 0.01	0.172 ± 0.01	0.252 ± 0.01
	Soil organic carbon (g·kg^−1^)	1.574 ± 0.10	1.966 ± 0.04	73.808 ± 1.4	85.646 ± 1.22	35.428 ± 1.02	64.002 ± 0.44
	Total nitrogen (g·kg^−1^)	0.182 ± 0.01	0.258 ± 0.01	5.376 ± 0.05	5.83 ± 0.10	3.382 ± 0.16	4.234 ± 0.073
	Total phosphorus (g·kg^−1^)	0.278 ± 0.02	0.376 ± 0.01	1.076 ± 0.04	1.112 ± 0.05	0.806 ± 0.06	1.144 ± 0.01

The test sites were strategically positioned within the designated national fixed monitoring sites, each encompassing an area of approximately 3 ha. Two treatments were established in temperate desert (TD), temperate steppe (TS), and mountain meadow (MM), respectively: grazing exclusion (GE) and free grazing (FG). Notably, all GE plots have been enclosed since 2012 and had remained enclosed for 12 years by the sampling year of 2024. The overall stocking rate for the three grassland sites ranged from 0.90 to 4.50 sheep units per hectare. The grazing intensities at these three sites fell within the category of moderate grazing in the local context, aligning with the carrying capacity of livestock in the area. Before the implementation of grazing exclusion, the areas designated for GE exhibited comparable vegetation composition, community characteristics, and landforms to the FG plots.

### 2.2 Plant and soil sampling and determination

**Figure 1:**
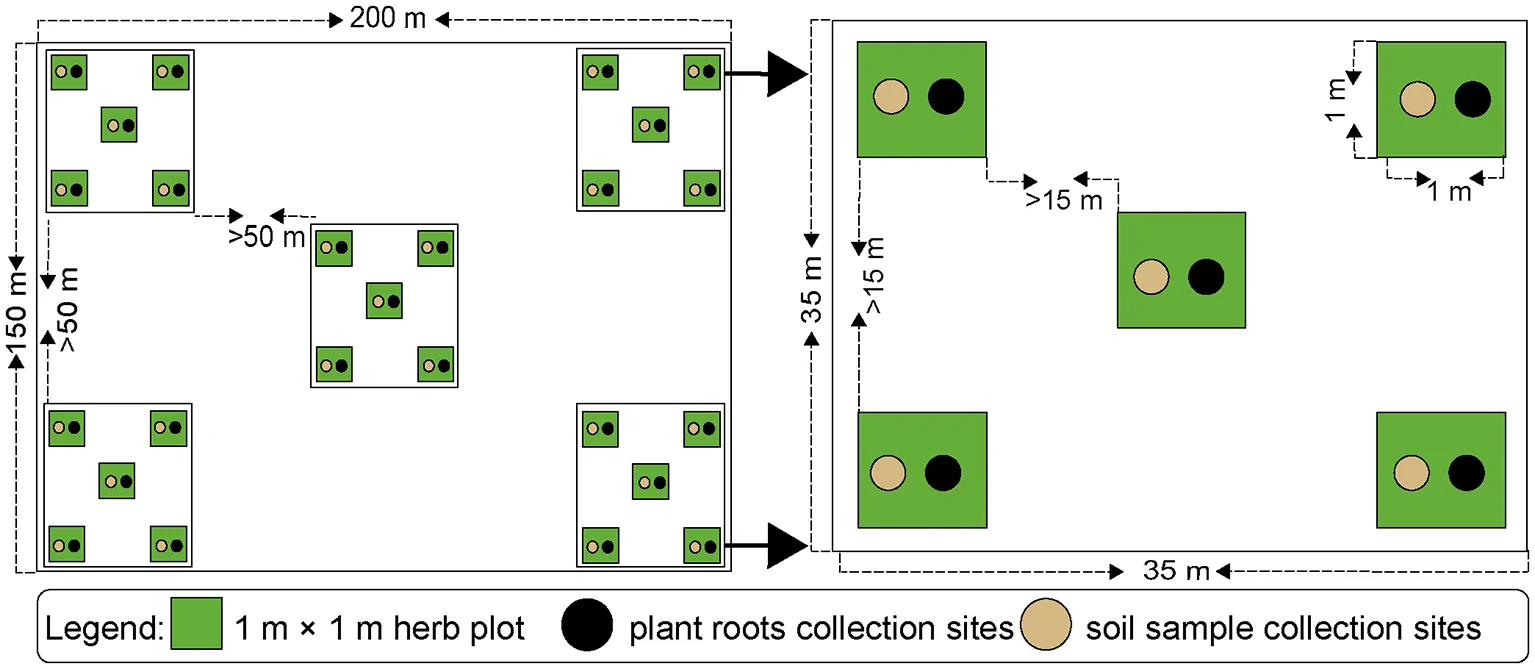
Sampling plot layout of a single treatment (identical for grazing exclusion and free grazing).

Field investigations were conducted in late June 2024. In each GE and corresponding FG area, five large plots (35 × 35 m each) were established with an interval of more than 50 m (Figure 1). Within each plot, five 1 × 1 m quadrats were arranged according to the five-point sampling method, with a distance of approximately 15 m between quadrats, resulting in a total of 150 quadrats sampled. The five plots established within each treatment area were considered spatial sampling units used to characterize within-treatment variability rather than independent grazing manipulations. In each quadrat, plant species were recorded, and vegetation characteristics, including coverage (%), height (cm), density (plants·m^−2^), and biomass, were measured. Above-ground biomass (AGB) was harvested, oven-dried at 105 °C for 30 min and subsequently at 80 °C for 24 h, and weighed. Below-ground biomass (BGB) was determined from intact soil blocks (10 × 20 cm) collected from each quadrat. The soil blocks were gently washed with water through a 2-mm mesh sieve to remove adhering soil particles. The recovered roots were manually cleaned to remove residual soil and visible debris, oven-dried at 80 °C for approximately 24 h until constant weight, and weighed to determine BGB, following Liu et al. (2022).

For soil sampling, a soil auger was used to collect samples from the 0–10 cm soil layer within each vegetation quadrat. The five subsamples collected from the five quadrats within the same plot were thoroughly mixed to form one composite sample per plot. Each composite sample was sealed in a labeled bag and transported to the laboratory. One subset was stored at −20 °C for microbial analysis, while the remaining samples were air-dried, sieved (2, 1, and 0.25 mm), and stored for physicochemical analyses. Thus, five plot-level samples were obtained for each treatment area and were used to characterize spatial variation within each grazing treatment.

Soil pH (soil:water = 5:1) and soil water content (SWC) were measured electrometrically and by gravimetry, respectively. Soil organic carbon (SOC), total nitrogen (TN), and total phosphorus (TP) were determined by potassium dichromate heating, Kjeldahl digestion, and molybdenum–antimony colorimetry, respectively. Microbial biomass carbon (MBC), nitrogen (MBN), and phosphorus (MBP) were determined using the chloroform fumigation–extraction method (Brookes et al., 1982, 1985; Vance et al., 1987). Briefly, fresh soil samples were divided into fumigated and non-fumigated subsamples, extracted with 0.5 M K_2_SO_4_ solution, and the differences between fumigated and non-fumigated extracts were used to calculate microbial biomass C, N, and P. The non-fumigated extracts obtained during the chloroform fumigation–extraction procedure were also used for the determination of soil dissolved organic carbon (DOC), nitrogen (DON), and phosphorus (DOP), which were subsequently incorporated into the CUE calculation. All datasets are original.

### 2.3 Determination of soil extracellular enzyme activities

Within the surveyed grassland community quadrats, soil microbial samples were collected from the 0–10 cm depth layer (Figure 1). Samples from each transect were thoroughly mixed, placed in sealed bags, and stored in a portable freezer at −20 °C for transport to the laboratory. Soil alkaline phosphatase (ALP), β-1,4-glucosidase (BG), leucine aminopeptidase (LAP), and β-1,4-N-acetylglucosaminidase (NAG) activities were measured using commercial assay kits (Nanjing Jice Biological Technology Co., Ltd., Nanjing, China) following the manufacturer's instructions. All soil samples were air-dried and sieved through a 0.5 mm mesh before analysis.

#### 2.3.1 Alkaline phosphatase (ALP)

Briefly, 0.1 g of air-dried soil was mixed with 50 μL toluene and incubated at room temperature for 15 min. Then, 0.4 mL of substrate solution (reagent 1) was added, and the mixture was incubated at 37 °C for 24 h. The reaction was terminated by adding 1 mL of reagent 2, followed by centrifugation at 8000 g for 10 min. The absorbance of the supernatant was measured at 660 nm using a microplate reader. One unit of ALP activity was defined as the amount of enzyme releasing 1 μmol of phenol per gram of dry soil per day (μmol d^−1^ g^−1^).

#### 2.3.2 β-1,4-Glucosidase (BG), leucine aminopeptidase (LAP), and β-1,4-N-acetylglucosaminidase (NAG)

For BG and NAG, 0.02 g of air-dried soil was mixed with 10 μL toluene and incubated for 15 min. Then, 130 μL of substrate solution (reagent 2) and 160 μL of buffer (reagent 3) were added, and the mixture was incubated at 37 °C for 1 h with shaking. The reaction was terminated by heating in a 90 °C water bath for 5 min, followed by centrifugation at 10,000 g for 10 min. The supernatant was then reacted with reagent 4 at room temperature for 2 min, and the absorbance was measured at 400 nm. For LAP, 0.05 g of fresh soil was mixed with 300 μL of reagent 1 and 300 μL of substrate solution (reagent 2), incubated at 37 °C for 1 h, and centrifuged at 8000 g for 10 min. The absorbance of the supernatant was measured at 405 nm. Standard curves were prepared using p-nitrophenol (for BG, NAG, and LAP) or phenol (for ALP). One unit of enzyme activity was defined as the amount of enzyme releasing 1 μmol of p-nitrophenol (or phenol for ALP) per gram of dry soil per day (μmol d^−1^ g^−1^).

### 2.4 Quantification of microbial metabolic limitations

Soil microbial resource limitations were assessed using vector analysis based on ecoenzymatic stoichiometry (Moorhead et al., 2016). The proportions of C-acquiring enzyme activity relative to P and N acquisition were calculated using Equations 1 and 2 as:


$$\begin{array}{l} \text{x}=\text{BG}/\left(\text{BG}+\text{AP}\right) \end{array}$$ (1)

$$\begin{array}{l} \text{y}=\text{BG}/\left(\text{BG}+\text{NAG}+\text{LAP}\right) \end{array}$$ (2)

where BG, NAG, LAP, and AP represent the activities of β-1,4-glucosidase, β-1,4-N-acetylglucosaminidase, leucine aminopeptidase, and alkaline phosphatase, respectively.

Vector length and vector angle were then calculated using Equations 3 and 4 as:


$$\begin{array}{l} \text{Vector length}=\text{SQRT}\left({\text{x}}^{2}+y^{2}\right) \end{array}$$ (3)

$$\begin{array}{l} \text{Vector angle}=\text{DEGREES}\left(\text{ATAN}2\left(\text{x},\text{y}\right)\right) \end{array}$$ (4)

Vector length indicates the degree of microbial carbon (C) limitation: a longer vector reflects stronger C limitation. Vector angle indicates the relative limitation between nitrogen (N) and phosphorus (P): angles < 45° indicate N limitation, while angles >45° indicate P limitation (Moorhead et al., 2016).

### 2.5 Estimation of microbial carbon use efficiency

Soil microbial carbon use efficiency (CUE) was estimated using the stoichiometric model based on the biogeochemical equilibrium approach (Sinsabaugh and Follstad Shah, 2012; Sinsabaugh et al., 2016). The calculations were performed as follows.

First, the scalars for nitrogen (N) and phosphorus (P) were calculated using Equations 5 and 6 as:


$$\begin{array}{l} {\text{S}}_{\text{C}:\text{N}}=\left(1/\text{EE}{\text{A}}_{\text{C}:\text{N}}\right)\times \left({\text{B}}_{\text{C}:\text{N}}/{\text{L}}_{\text{C}:\text{N}}\right) \end{array}$$ (5)

$$\begin{array}{l} {\text{S}}_{\text{C}:\text{P}}=\left(1/\text{EE}{\text{A}}_{\text{C}:\text{P}}\right)\times \left({\text{B}}_{\text{C}:\text{P}}/{\text{L}}_{\text{C}:\text{P}}\right) \end{array}$$ (6)

where the parameters in Equations 7–12 are defined as follows:


$$\begin{array}{l} \text{EE}{\text{A}}_{\text{C}:\text{N}}=\text{BG}/\left(\text{NAG}+\text{LAP}\right)\\ \;\times \left(\text{ratio of C- }\text{to N}\text{-acquiring enzyme activities}\right) \end{array}$$ (7)

$$\begin{array}{l} \text{EE}{\text{A}}_{\text{C}:\text{P}}=\text{BG}/\text{AP }\\ \;\times \left(\text{ratio of C- to P}\text{-acquiring enzyme activities}\right) \end{array}$$ (8)

$$\begin{array}{l} {\text{B}}_{\text{C}:\text{N}}=\text{MBC}/\text{MBN }\left(\text{microbial biomass C}:\text{N ratio}\right) \end{array}$$ (9)

$$\begin{array}{l} {\text{B}}_{\text{C}:\text{P}}=\text{MBC}/\text{MBP }\left(\text{microbial biomass C}:\text{P ratio}\right) \end{array}$$ (10)

$$\begin{array}{l} {\text{L}}_{\text{C}:\text{N}}=\text{DOC}/\text{DON }\left(\text{dissolved organic C}:\text{N ratio}\right) \end{array}$$ (11)

$$\begin{array}{l} {\text{L}}_{\text{C}:\text{P}}=\text{DOC}/\text{DOP }\left(\text{dissolved organic C}:\text{P ratio}\right) \end{array}$$ (12)

BG, NAG, LAP, and AP represent the activities of β-1,4-glucosidase, β-1,4-N-acetylglucosaminidase, leucine aminopeptidase, and alkaline phosphatase, respectively. MBC, MBN, and MBP are microbial biomass carbon, nitrogen, and phosphorus; DOC, DON, and DOP are dissolved organic carbon, nitrogen, and phosphorus.

Then, microbial CUE was computed using Equation 13 as:


$$\begin{array}{l} \text{CUE}={\text{CUE}}_{\text{max}}\times [({\text{S}}_{\text{C}:\text{N}}\times {\text{S}}_{\text{C}:\text{P}})\text{/}(({\text{K}}_{\text{C}:\text{N}}{\text{+S}}_{\text{C}:\text{N}})\\ \;\times ({\text{K}}_{\text{C}:\text{P}}{\text{+S}}_{\text{C}:\text{P}})){]}^{\text{1/2}} \end{array}$$ (13)

Following previous theoretical and empirical studies based on the biogeochemical equilibrium model (Sinsabaugh and Follstad Shah, 2012; Sinsabaugh et al., 2016), the maximum carbon use efficiency (CUEmax) was set to 0.6, and the half-saturation constants KC:N and KC:P were both set to 0.5. These fixed parameters were applied consistently across all grassland ecosystems to allow comparisons of relative changes in microbial CUE under different grazing regimes.

### 2.6 Data statistics and analysis

Data preprocessing was performed using Microsoft Excel 2020 (Microsoft Corporation, Redmond, WA, USA). All statistical analyses were conducted using R 4.5.3 (R Core Team, 2025) and IBM SPSS Statistics 25.0 (IBM Corporation, Armonk, NY, USA). Results are presented as mean ± standard error (SE), and statistical significance was set at *P* < 0.05 unless otherwise stated.

First, differences associated with grazing exclusion (GE), grassland type (TD, TS, and MM), and their interaction on soil variables (dissolved organic nutrients, microbial biomass, enzyme activities, vector characteristics, and CUE) were evaluated using two-way analysis of variance (ANOVA) in SPSS. Because soil samples were collected at the plot level, each plot-level sample was treated as a spatial sampling unit representing within-treatment variation. Before ANOVA, normality of residuals was checked using the Shapiro–Wilk test, and homogeneity of variances was assessed using Levene's test (α = 0.05). When significant interactions were detected, Tukey's HSD *post-hoc* test was applied for multiple comparisons among treatment groups. Independent-sample *t*-tests were used to compare grazing exclusion and freely grazed areas within each grassland type, whereas one-way ANOVA followed by Duncan's multiple range test was used to evaluate differences among grassland types under the same grazing condition. For variables violating normality or homoscedasticity assumptions, data were log-transformed or analyzed using the non-parametric Kruskal–Wallis test.

Second, to explore relationships among plant traits (AGB, BGB, and Shannon–Wiener index), soil properties (pH, SWC, SOC, TN, and TP), dissolved organic nutrients (DOC, DON, and DOP), microbial biomass (MBC, MBN, and MBP), enzyme activities (BG, NAG+LAP, and AP), vector characteristics (length and angle), and microbial CUE, Spearman correlation analysis was performed in R using the ‘corrplot' package. Correlation matrices were visualized as heatmaps, and significant correlations (*P* < 0.05) were considered in subsequent analyses.

Third, to identify key predictors associated with microbial vector characteristics (vector length and angle) and CUE, random forest (RF) analysis was conducted using the ‘randomForest' and ‘rfPermute' packages in R. The importance of each predictor was evaluated using the percentage increase in mean squared error (%IncMSE). RF models were constructed separately for each grassland type (TD, TS, and MM), with 5000 regression trees. The number of predictors randomly selected at each split was set according to the square root of the total number of candidate predictors. A fixed random seed (123) was used to ensure reproducibility. The RF analysis was used to identify variables showing relatively high predictive importance for microbial vector characteristics and CUE. The statistical significance of variable importance was assessed using permutation tests implemented in the ‘rfPermute' package, with 100 permutation replicates. These results were considered together with significant Spearman correlations to determine candidate variables for subsequent PLS-SEM analysis.

Fourth, to examine the hypothesized association pathways linking grazing exclusion, plant characteristics, soil properties, dissolved organic nutrients, microbial biomass, microbial nutrient limitation, and CUE, partial least squares path modeling (PLS-PM) was performed using the ‘plspm' package (v0.4.9) in R. Separate PLS-PM models were constructed for each grassland type (TD, TS, and MM) to characterize ecosystem-specific association pathways.

Variables included in the PLS-PM models were selected based on ecological hypotheses, RF variable importance, and significant Spearman correlations, while avoiding highly redundant variables. Selected observed indicators were grouped into ecologically meaningful latent constructs according to their ecological functions. Plant characteristics were represented by selected plant indicators, soil properties by selected physicochemical indicators, dissolved organic nutrients by selected dissolved nutrient indicators, and microbial biomass by selected microbial biomass indicators. Grazing exclusion (GE) was coded as a binary exogenous variable (0 = freely grazed, 1 = grazing exclusion), whereas vector length, vector angle, and microbial CUE were specified as endogenous variables.

All latent constructs were specified using a reflective measurement model (Mode A). The structural model was established according to hypothesized ecological relationships among these constructs. Path coefficients were estimated using the PLS algorithm with standardized variables and the centroid weighting scheme. No *post hoc* modification of model pathways was conducted; all model structures were specified *a priori* based on ecological hypotheses and statistical screening results.

Model evaluation included indicator loadings, Dillon–Goldstein's rho (DG.rho), average variance extracted (AVE), coefficient of determination (*R*^2^), and goodness-of-fit (GoF). Predictive relevance based on blindfolding (*Q*^2^) was not assessed because this procedure is not implemented in the ‘plspm' package used in this study.

The structural model was established according to ecological hypotheses regarding grazing exclusion effects on plant–soil–microbe interactions and microbial nutrient limitation. Grazing exclusion (GE, coded as 0/1) was specified as an exogenous variable, whereas vector length (C limitation), vector angle (N vs. P limitation), and microbial CUE were specified as endogenous variables. Path coefficients were estimated using the PLS algorithm. Model performance was assessed using *goodness-of-fit* (GoF), coefficient of determination (*R*^2^), indicator loadings, composite reliability (DG.rho), and average variance extracted (AVE). No *post hoc* model modification was conducted; all pathways were established based on ecological hypotheses and statistical screening results.

All statistical graphics were programmatically generated using the ‘ggplot2′ package (v3.4.2) in R 4.5.3, with subsequent vector refinement and compositional optimization performed in Adobe Illustrator 2021 (Adobe Inc.). Vector length and angle calculations, as well as CUE estimation, followed the procedures described in Sections 2.4 and 2.5. Data are available from the corresponding author upon reasonable request.

## 3 Results

### 3.1 Effects of grazing exclusion on soil dissolved organic nutrients and microbial biomass

**Figure 2:**
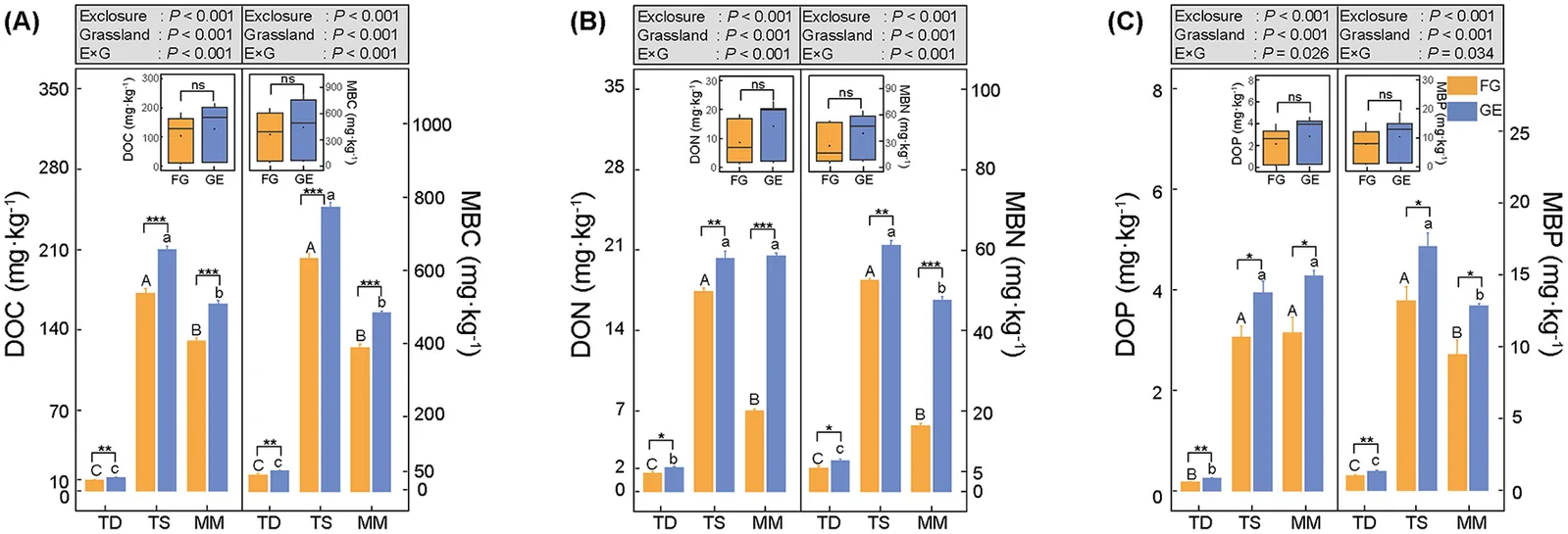
Effects of grazing exclusion on soil dissolved organic nutrients and microbial biomass across three grassland types. **(A)** Soil dissolved organic carbon (DOC) and microbial biomass carbon (MBC); **(B)** Soil dissolved organic nitrogen (DON) and microbial biomass nitrogen (MBN); **(C)** Soil dissolved organic phosphorus (DOP) and microbial biomass phosphorus (MBP). Data are presented as mean ± standard error (SE). “*,” “**,” and “***' indicate significant differences between grazing exclusion and freely grazing treatments within the same grassland type at *P* < 0.05, *P* < 0.01, and *P* < 0.001, respectively, as determined by independent-samples t-tests, whereas “ns” indicates no significant difference (*P* > 0.05). Capital letters indicate significant differences among freely grazing areas of different grassland types (*P* < 0.05), whereas lowercase letters indicate significant differences among grazing exclusion areas of different grassland types (*P* < 0.05), as determined by one-way ANOVA followed by Duncan's multiple range test; shared letters indicate no significant difference (*P* > 0.05). TD, temperate desert; TS, temperate steppe; MM, mountain meadow; GE, grazing exclusion plots; FG, freely grazing plots. Gray boxes above the bar charts show the results of two-way ANOVA for grazing exclusion, grassland type, and their interaction. Box plots show the combined effects of grazing exclusion across the three grassland types as determined by one-way ANOVA followed by Duncan's multiple range test.

Grazing exclusion significantly altered the contents of dissolved organic carbon, nitrogen, and phosphorus (DOC, DON, and DOP) across the three grassland types (Figure 2). Overall, the concentrations of DOC, DON, and DOP increased significantly after grazing exclusion in all grasslands (*P* < 0.05), with increments ranging from 16.54 to 191.25% (Figures 2A–C). Similarly, soil microbial biomass (MBC, MBN, and MBP) also increased significantly following grazing exclusion, with increments ranging from 16.46 to 191.17% (*P* < 0.05; Figures 2A–C).

To further elucidate the effects of grazing exclusion, grassland type, and their interaction on soil dissolved organic nutrients and microbial biomass, we performed a two-way analysis of variance (ANOVA). Regarding the main effects, both grazing exclusion and grassland type exerted highly significant effects on the concentrations of DOC, DON, DOP, MBC, MBN, and MBP (*P* < 0.001). More importantly, significant interactive effects between grazing exclusion and grassland type were detected for all these variables (*P* < 0.05; Figures 2A–C), indicating the complexity of grazing exclusion effects across different grassland ecosystems.

### 3.2 Effects of grazing exclusion on the activities of key enzymes involved in soil C, N, and P cycling

**Figure 3:**
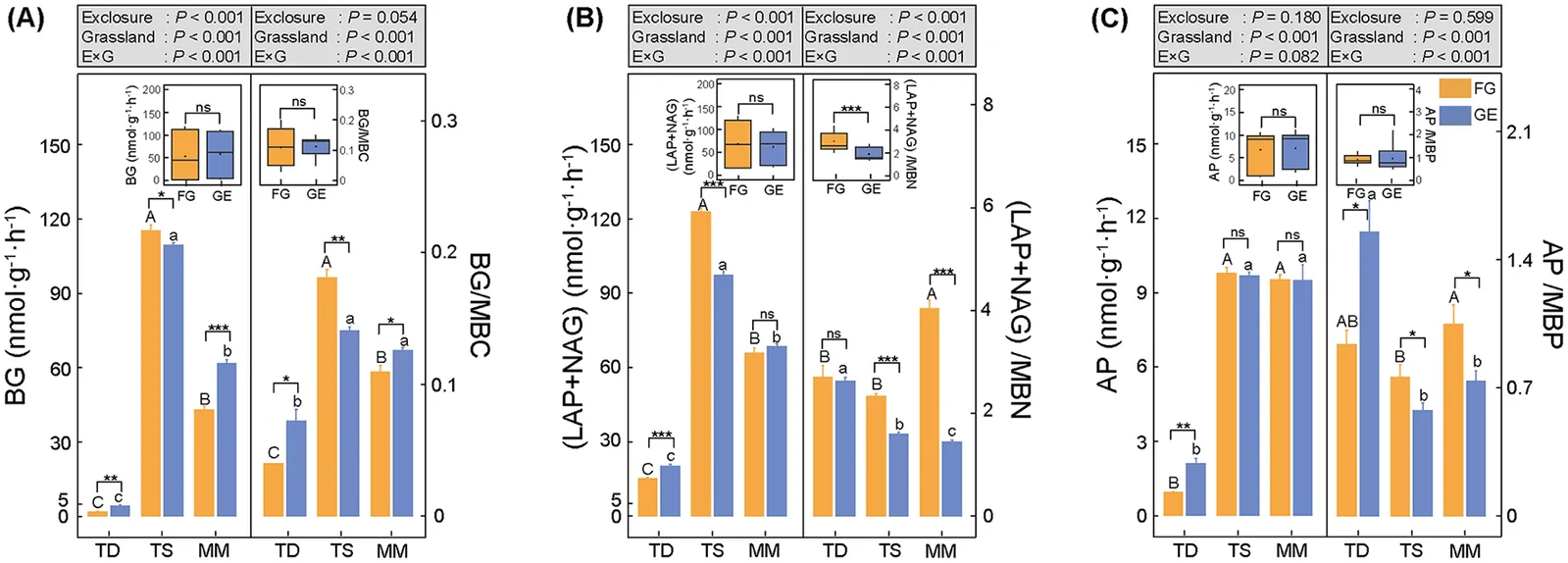
Responses of soil C-, N-, and P-acquiring enzyme activities to grazing exclusion across three grassland types. **(A)** Soil C-acquiring enzyme (BG) activity and its ratio to microbial biomass carbon (BG/MBC); **(B)** Soil N-acquiring enzyme (LAP+NAG) activities and their ratio to microbial biomass nitrogen ((LAP+NAG)/MBN); **(C)** Soil P-acquiring enzyme (AP) activity and its ratio to microbial biomass phosphorus (AP/MBP). Data are presented as mean ± standard error (SE). “*,” “**,” and “***” indicate significant differences between grazing exclusion and freely grazing treatments within the same grassland type at *P* < 0.05, *P* < 0.01, and *P* < 0.001, respectively, as determined by independent-samples t-tests, whereas “ns” indicates no significant difference (*P* > 0.05). Capital letters indicate significant differences among freely grazing areas of different grassland types (*P* < 0.05), whereas lowercase letters indicate significant differences among grazing exclusion areas of different grassland types (*P* < 0.05), as determined by one-way ANOVA followed by Duncan's multiple range test; shared letters indicate no significant difference (*P* > 0.05). TD, temperate desert; TS, temperate steppe; MM, mountain meadow; GE, grazing exclusion plots; FG, freely grazing plots. Gray boxes above the bar charts show the results of two-way ANOVA for grazing exclusion, grassland type, and their interaction. Box plots show the combined effects of grazing exclusion across the three grassland types as determined by one-way ANOVA followed by Duncan's multiple range test.

To investigate the effects of grazing exclusion on the activities of key enzymes involved in soil carbon (C), nitrogen (N), and phosphorus (P) acquisition, we measured the activities of β-1,4-glucosidase (BG), the sum of leucine aminopeptidase and β-1,4-N-acetylglucosaminidase (LAP+NAG), and alkaline phosphatase (AP) across three grassland types (Figure 3).

In temperate desert (TD), grazing exclusion significantly increased the activities of all three enzymes, with increments ranging from 31.47 to 123.85% (*P* < 0.01; Figures 3A–C). In contrast, in temperate steppe (TS), enzyme activities decreased to varying degrees after grazing exclusion, with BG and (LAP+NAG) activities significantly reduced by 5.01 and 20.72%, respectively (*P* < 0.05; Figures 3A–C). In mountain meadow (MM), only BG activity increased significantly (by 43.65%) following grazing exclusion (*P* < 0.001; Figure 3A).

We further calculated the ecoenzymatic coefficients (i.e., the proportions of C-, N-, and P-acquiring enzyme activities relative to their total activity). In TD, the C- and P-acquiring enzyme coefficients increased significantly after grazing exclusion, by 80.50 and 65.58%, respectively (*P* < 0.05; Figures 3A, C). In TS, all three coefficients decreased significantly, with reductions ranging from 22.39 to 31.86% (*P* < 0.05; Figures 3A–C). In MM, the C-acquiring coefficient increased significantly by 15.08% (*P* < 0.05; Figure 3A), while the N- and P-acquiring coefficients decreased significantly by 64.36 and 29.51%, respectively (*P* < 0.05; Figures 3B, C).

Two-way ANOVA revealed that, for main effects, grazing exclusion had highly significant effects on BG, (LAP+NAG), and (LAP+NAG)/MBN (*P* < 0.001); grassland type had highly significant effects on all measured variables (*P* < 0.001). More importantly, the interaction between grazing exclusion and grassland type exerted highly significant effects on all variables except AP (*P* < 0.001; Figures 3A–C), indicating that grazing exclusion effects differed among contrasting grassland ecosystems with distinct environmental backgrounds.

### 3.3 Effects of grazing exclusion on soil enzyme vector characteristics

**Figure 4:**
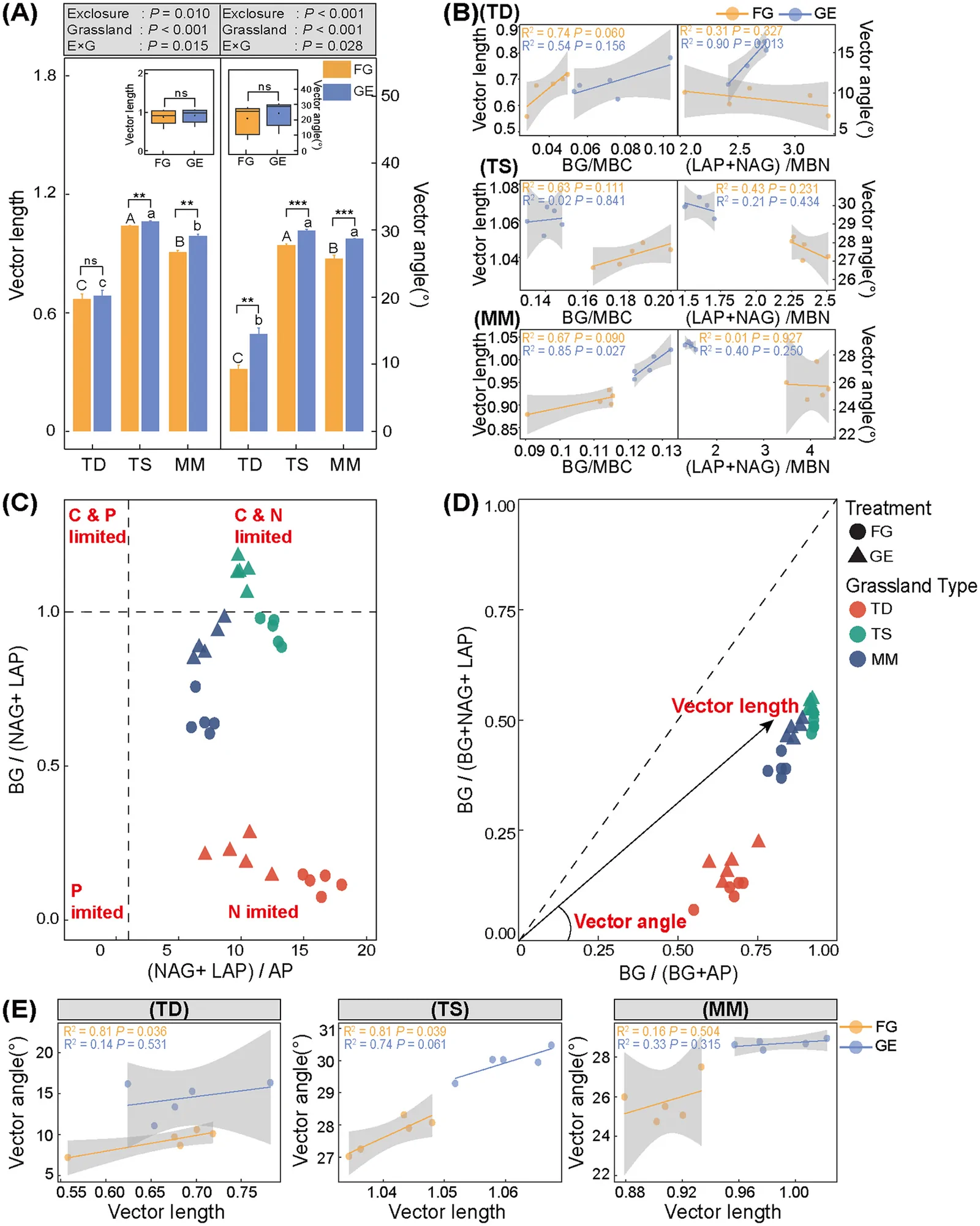
Vector characteristics of soil ecoenzymatic stoichiometry across three grassland types. **(A)** Vector length and angle. Data are presented as mean ± standard error (SE). “*,” “**,” and “***” indicate significant differences between grazing exclusion and freely grazing treatments within the same grassland type at *P* < 0.05, *P* < 0.01, and *P* < 0.001, respectively, as determined by independent-samples t-tests, whereas “ns” indicates no significant difference (*P* > 0.05). Capital letters indicate significant differences among freely grazing areas of different grassland types (*P* < 0.05), whereas lowercase letters indicate significant differences among grazing exclusion areas of different grassland types (*P* < 0.05), as determined by one-way ANOVA followed by Duncan's multiple range test; shared letters indicate no significant difference (*P* > 0.05). In **(A)**, gray boxes above the bar charts show the results of two-way ANOVA for grazing exclusion, grassland type, and their interaction. Box plots show the combined effects of grazing exclusion across the three grassland types as determined by one-way ANOVA followed by Duncan's multiple range test. **(B)** Linear regressions between enzyme activity ratios and vector characteristics. Shaded areas indicate 95% confidence intervals; *R*^2^ and P-values are shown for each regression. **(C)** Scatter plot of ecoenzymatic ratios. **(D)** Relative microbial metabolic limitation. **(E)** Relationship between vector length and angle under grazing exclusion across the three grassland types. TD, temperate desert; TS, temperate steppe; MM, mountain meadow; GE, grazing exclusion plots; FG, freely grazing plots.

The vector characteristics (length and angle) of soil ecoenzymatic stoichiometry are shown in Figure 4A. Regarding vector length: grazing exclusion increased vector length in all three grassland types, with significant increases in TS and MM by 1.83 and 8.71%, respectively (*P* < 0.01), indicating that microbial C limitation was progressively aggravated after grazing exclusion. Regarding vector angle: vector angles were consistently below 45° across all treatments, indicating that microbial communities were always limited by N. After grazing exclusion, vector angles increased significantly in all three grasslands, by 55.74%, 8.08%, and 11.37%, respectively (*P* < 0.01), suggesting that N limitation gradually weakened (Figure 4A).

To explore the relationships between C-/N-acquiring enzyme coefficients and vector characteristics, we performed linear regression analyses between C-acquiring coefficient and vector length, and between N-acquiring coefficient and vector angle, separately for each grassland type (Figure 4B). In TD, the explanatory power of the regression model for C-acquiring coefficient vs. vector length decreased after grazing exclusion, whereas that for N-acquiring coefficient vs. vector angle increased and became significant (*P* < 0.05). In TS, the explanatory power of both regression models decreased after grazing exclusion, and neither was significant (*P* > 0.05). In MM, the explanatory power of both regression models increased after grazing exclusion, and the model for C-acquiring coefficient vs. vector length reached significance (*P* < 0.05).

The scatter plot (Figure 4C) shows the microbial nutrient limitation patterns revealed by ecoenzymatic stoichiometry (BG/(LAP+NAG) vs. (LAP+NAG)/AP). Across all treatments, soil microbial communities in TD and MM were N-limited, while those in TS experienced co-limitation by C and N after grazing exclusion. The relative microbial metabolic limitation derived from soil ecoenzymatic stoichiometry (EES) further confirmed that all data points under grazing exclusion fell below the 1:1 line (vector angle < 45°), indicating strong N limitation (Figure 4D), consistent with the above results.

Furthermore, linear regression analysis showed that vector length and vector angle were positively correlated in all three grassland types after grazing exclusion, although none of the correlations reached statistical significance (*P* > 0.05). This correlation suggests a dynamic coupling between microbial C limitation and N limitation, which exhibited an inverse relationship, i.e., enhanced C limitation accompanied by weakened N limitation (Figure 4E).

### 3.4 Effects of grazing exclusion on soil microbial carbon use efficiency

**Figure 5:**
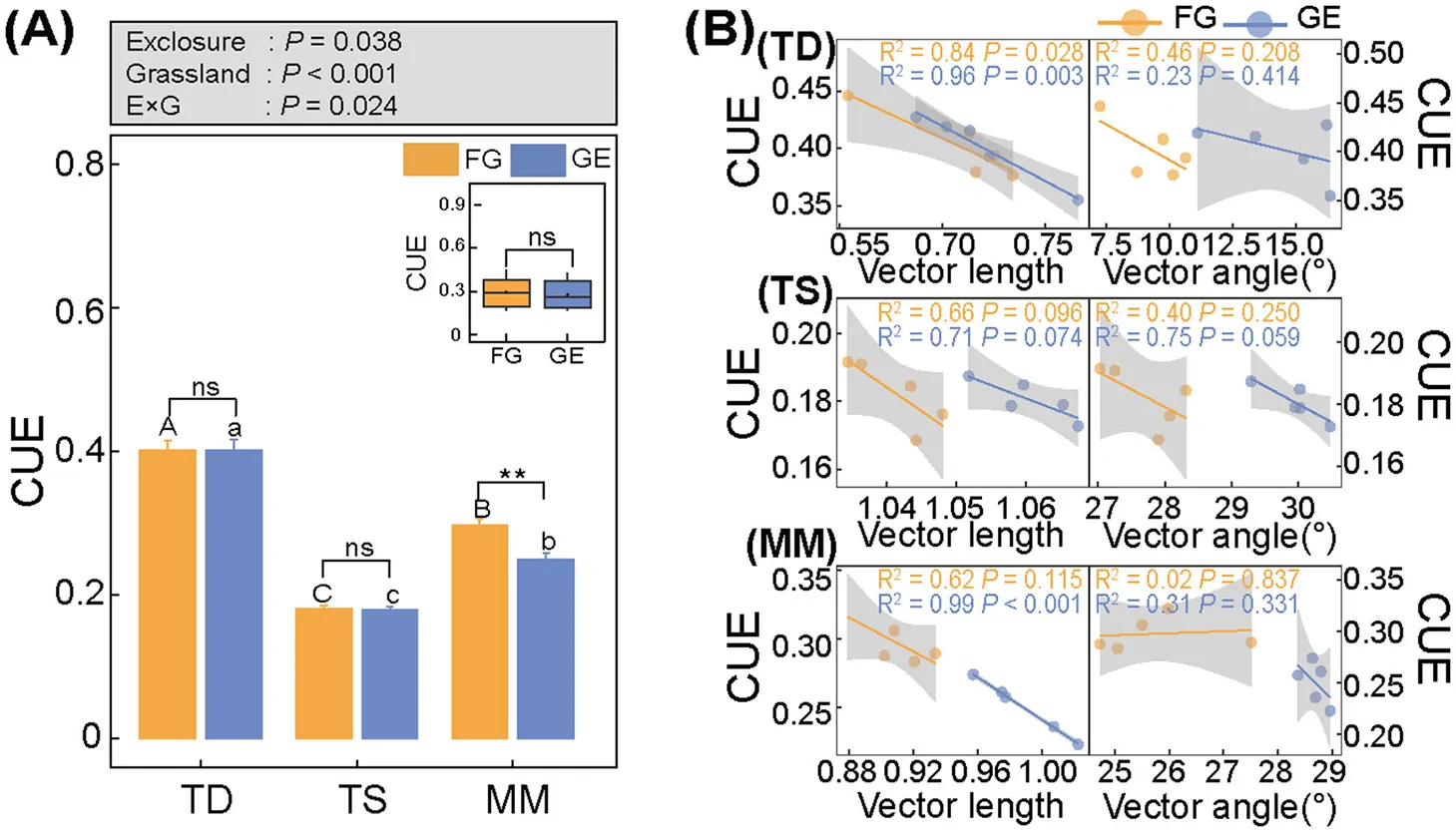
Effects of grazing exclusion on soil microbial carbon use efficiency across three grassland types. (A) Microbial carbon use efficiency (CUE) across the three grassland types. Data are presented as mean ± standard error (SE). “*,” “**,” and “***” indicate significant differences between grazing exclusion and freely grazing treatments within the same grassland type at *P* < 0.05, *P* < 0.01, and *P* < 0.001, respectively, as determined by independent-samples t-tests, whereas “ns” indicates no significant difference (*P* > 0.05). Capital letters indicate significant differences among freely grazing areas of different grassland types (*P* < 0.05), whereas lowercase letters indicate significant differences among grazing exclusion areas of different grassland types (*P* < 0.05), as determined by one-way ANOVA followed by Duncan's multiple range test; shared letters indicate no significant difference (*P* > 0.05). Gray boxes above the bar charts show the results of two-way ANOVA for grazing exclusion, grassland type, and their interaction. Box plots show the combined effects of grazing exclusion across the three grassland types as determined by one-way ANOVA followed by Duncan's multiple range test. **(B)** Linear relationships between vector characteristics (vector length and vector angle) and CUE across the three grassland types. Shaded areas indicate 95% confidence intervals; *R*^2^ and P-values are shown for each regression. TD, temperate desert; TS, temperate steppe; MM, mountain meadow; GE, grazing exclusion plots; FG, freely grazing plots.

The effects of grazing exclusion on soil CUE across the three grassland types are shown in Figure 5A. After grazing exclusion, CUE significantly decreased only in the mountain meadow (MM), by 15.92% (*P* < 0.01; Figure 5A).

Linear regression analyses between vector length, vector angle, and CUE were performed separately for each grassland type (Figure 5B). Overall, both vector length and vector angle exhibited negative correlations with CUE across the three grasslands. Specifically, in TD, the explanatory power of the regression model for vector length vs. CUE increased after grazing exclusion and reached a highly significant level (*P* < 0.01). In TS, the explanatory power of the regression models for both vector length vs. CUE and vector angle vs. CUE increased, but neither was significant (*P* > 0.05). In MM, the trends were similar to those in TS, but the explanatory power of the vector length vs. CUE regression model increased and reached a highly significant level (*P* < 0.001).

### 3.5 Correlations and key predictors of vector characteristics and CUE

**Figure 6:**
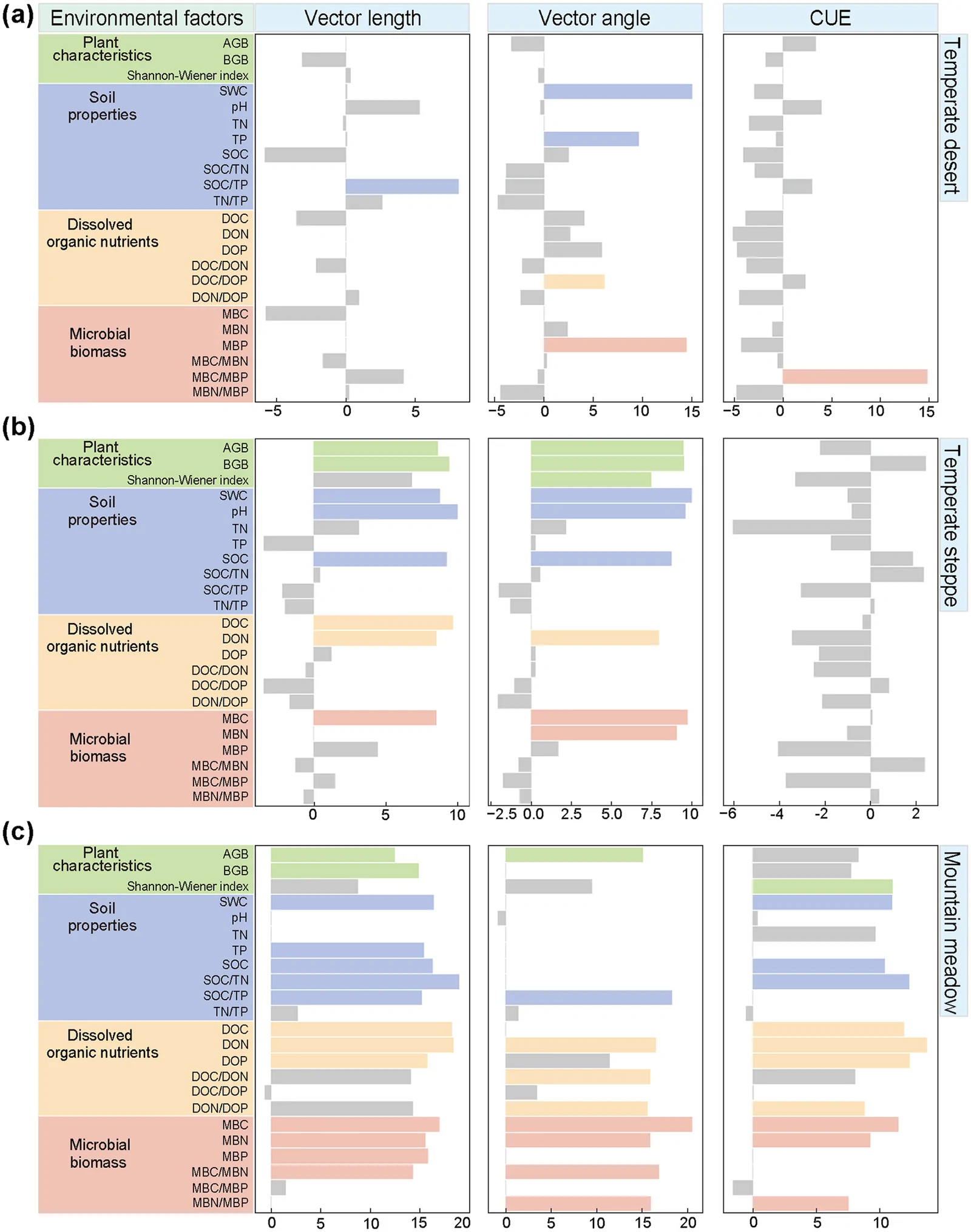
Random forest analysis identifying key predictors of vector length, vector angle, and CUE across the three grassland types. **(A)** Temperate desert. **(B)** Temperate steppe. **(C)** Mountain meadow. Bar length represents the percentage increase in mean squared error (%IncMSE), indicating the relative importance of each predictor. Colored bars indicate predictors with significant permutation-based importance (*P* < 0.05), whereas gray bars indicate non-significant predictors (*P* > 0.05). Green, blue, orange, and red bars represent plant characteristics, soil properties, dissolved organic nutrients, and microbial biomass, respectively. CUE, microbial carbon use efficiency.

To further elucidate the mechanisms underlying the effects of grazing exclusion on soil ecoenzymatic stoichiometric vector characteristics and CUE, we employed two complementary statistical approaches: Spearman correlation analysis and random forest modeling. Correlation analysis was first used to evaluate the relationships between plant traits, soil physicochemical properties, dissolved organic nutrients, microbial biomass, and vector characteristics as well as CUE (Supplementary Figure A3). Subsequently, random forest models were applied to identify the key predictors driving vector characteristics and CUE (Figure 6).

TD: correlation analysis revealed that the only vector angle was significantly correlated with environmental factors. Specifically, SWC, TN, TP, DOC, DON, DOP, MBN, and MBP showed significant positive correlations with vector angle (*P* < 0.05), whereas BGB exhibited a significant negative correlation (*P* < 0.01; Supplementary Figure A3). Random forest results indicated that the key predictor for vector length was SOC/TP; for vector angle, SWC, TP, DOC/DOP, and MBP; and for CUE, only MBC/MBP (Figure 6A).

TS: except for CUE, which showed no significant correlation with any environmental factor, both vector length and vector angle were significantly correlated with multiple factors. Specifically, AGB, BGB, Shannon–Wiener index, SOC, DOC, DON, MBC, and MBN were significantly positively correlated with both vector length and vector angle (*P* < 0.05), whereas pH showed a significant negative correlation (*P* < 0.01; Supplementary Figure A3). Random forest models yielded similar results, with the key predictors for vector length and vector angle being AGB, BGB, SWC, pH, SOC, DON, and MBC (Figure 6B).

MM: most variables (AGB, BGB, Shannon–Wiener index, SWC, TN, TP, SOC, SOC/TN, SOC/TP, DOC, DON, DOP, DON/DOP, MBC, MBN, MBP, MBN/MBP) were significantly positively correlated with vector length and vector angle (*P* < 0.05), but significantly negatively correlated with CUE (*P* < 0.05). In contrast, DOC/DON and MBC/MBN showed the opposite pattern: significantly negative correlations with vector characteristics and significantly positive correlations with CUE (*P* < 0.05; Supplementary Figure A3). Random forest results indicated that the key predictors for vector length included AGB, BGB, SWC, TP, SOC, SOC/TN, SOC/TP, DOC, DON, DOP, MBC, MBN, MBP, and MBC/MBN; for vector angle, AGB, SOC/TP, DON, DOC/DON, DON/DOP, MBC, MBN, MBC/MBN, and MBN/MBP; and for CUE, the Shannon–Wiener index, SWC, SOC, SOC/TN, DOC, DON, DOP, DON/DOP, MBC, MBN, and MBN/MBP (Figure 6C).

### 3.6 PLS-SEM analysis of microbial C/N limitation and CUE under grazing exclusion

**Figure 7:**
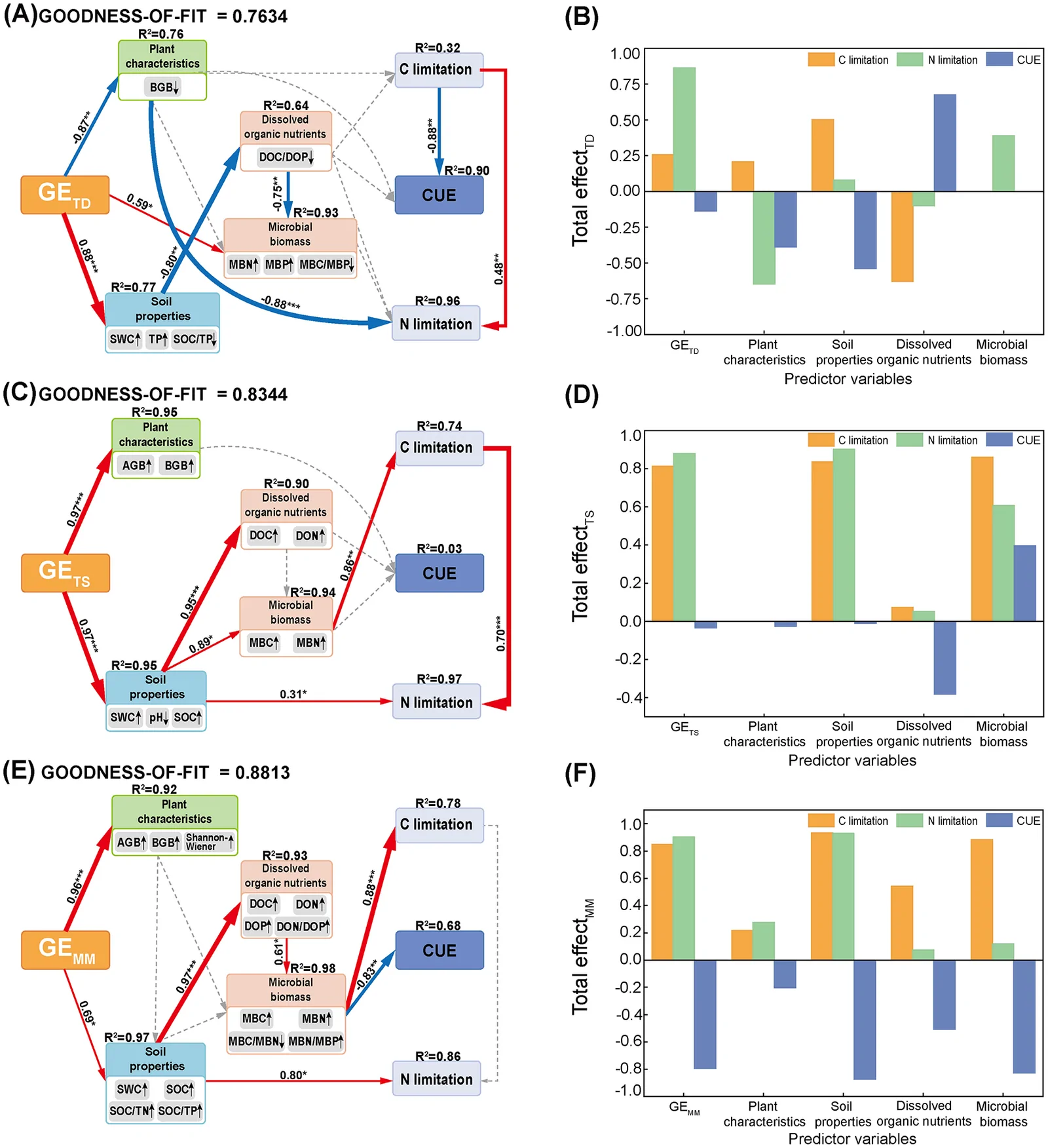
Associations among grazing exclusion, soil microbial C and N limitations, and CUE across three grassland types. **(A, C, E)** Partial least squares-path models (PLS-PM) for temperate desert, temperate steppe, and mountain meadow, respectively. **(B, D, F)** Standardized total effects of the environmental factors on microbial C limitation, N limitation, and CUE for each grassland type. Path coefficients are shown on the arrows. Solid red and blue arrows represent significant positive and negative associations, respectively, with line thickness proportional to the significance level (*P < 0.05, **P < 0.01, ***P < 0.001). Non-significant associations are shown as thin gray dashed lines. *R*^2^ values indicate the proportion of variance explained by the corresponding endogenous variable. GE, grazing exclusion; TD, temperate desert; TS, temperate steppe; MM, mountain meadow; CUE, microbial carbon use efficiency.

Partial least squares structural equation modeling (PLS-SEM) was employed to explore the potential pathways associated with grazing exclusion, soil microbial resource limitations (vector characteristics), and carbon use efficiency (CUE) across the three grassland types (Figure 7).

TD: grazing exclusion was primarily negatively associated with plant belowground biomass (BGB), which was in turn associated with N limitation (path coefficient = −0.88, *P* < 0.001). Additionally, grazing exclusion was positively associated with soil properties (SWC, TP, and SOC/TP), which were associated with variation in dissolved organic nutrients (DOC/DOP) and, in turn, with C limitation (vector length, *R*^2^ = 0.32) and CUE (*R*^2^ = 0.90; Figure 7A). Total-effect estimates (Figure 7B) indicated that grazing exclusion, plant traits (BGB), and microbial biomass (MBN, MBP, and MBC/MBP) showed relatively strong associations with N limitation, whereas soil properties (SWC, TP, and SOC/TP) and dissolved organic nutrients (DOC/DOP) showed stronger associations with C limitation and CUE.

TS: grazing exclusion was directly and positively associated with soil properties (SWC, pH, and SOC), which were subsequently associated with N limitation (path coefficient = 0.31, *P* < 0.05). Meanwhile, grazing exclusion was indirectly associated with C limitation through its association with microbial biomass (MBC, MBN; path coefficient = 0.86, *P* < 0.01; Figure 7C). Total-effect estimates (Figure 7D) indicated that grazing exclusion, soil properties, and microbial biomass showed strong associations with both C and N limitation, while dissolved organic nutrients (DOC and DON) and microbial biomass showed relatively strong associations with CUE.

MM: similar to the temperate steppe, grazing exclusion was directly and positively associated with soil properties (SWC, SOC, SOC/TN, and SOC/TP), which were subsequently associated with N limitation (path coefficient = 0.80, *P* < 0.05). Furthermore, grazing exclusion was associated with variation in dissolved organic nutrients (DOC, DON, DOP, and DON/DOP), which was associated with microbial biomass (MBC, MBN, MBC/MBN, and MBN/MBP) and ultimately showed a positive association with C limitation (path coefficient = 0.88, *P* < 0.001) and a negative association with CUE (path coefficient = −0.83, *P* < 0.01; Figure 7E). Total-effect estimates (Figure 7F) indicated that grazing exclusion and soil properties showed relatively strong associations with N limitation, whereas soil properties and microbial biomass showed stronger associations with C limitation and CUE.

## 4 Discussion

### 4.1 Effects of grazing exclusion on soil enzyme activities and microbial carbon use efficiency

The present study revealed marked differences in the responses of soil C- (BG), N- (LAP+NAG), and P- (AP) acquiring enzyme activities to grazing exclusion among the three grassland types (Figure 3). In TD, GE significantly increased all three enzyme activities, with increments ranging from 31.47 to 123.85%. In TS, enzyme activities decreased to varying degrees under GE, with BG and (LAP+NAG) significantly reduced by 5.01 and 20.72%, respectively, while AP showed no significant change. In MM, only BG activity increased significantly under GE (by 43.65%), whereas (LAP+NAG) and AP remained unchanged. These grassland-type-specific responses indicate that the regulatory effect of grazing exclusion on enzyme activities is highly dependent on the background conditions of each grassland.

The changes in enzyme activities after grazing exclusion were closely associated with shifts in soil resource supply. Previous studies have demonstrated that vegetation restoration significantly increases the activities of C-, N-, and P-acquiring enzymes (Wang et al., 2020; Cui et al., 2018; Yang et al., 2020; Yao et al., 2023). In our study, grazing exclusion significantly increased soil dissolved organic carbon (DOC), nitrogen (DON), phosphorus (DOP), and microbial biomass carbon, nitrogen, and phosphorus (MBC, MBN, and MBP) across all three grasslands, with increments ranging from 16 to 191% (Figure 2). Notably, in TD, both aboveground and belowground biomass decreased after grazing exclusion, yet soil nutrients and microbial biomass increased significantly, and enzyme activities were greatly enhanced. This suggests that the increase in enzyme activities may depend mainly on aboveground litter input and the improvement of soil resource availability rather than on living plant biomass. In TS, although aboveground and belowground biomass increased substantially (AGB by 140%, BGB by 22%) and soil nutrients and microbial biomass also increased, enzyme activities declined, implying that other limiting factors (e.g., litter quality, plant-microbe competition intensity, or adjustments in microbial metabolic strategies) may play a dominant role. In MM, both AGB and BGB increased significantly (by 76 and 84%, respectively), and soil resources improved, but only C-acquiring enzyme activity increased, while N- and P-acquiring enzymes showed no significant change, indicating that microorganisms may prioritize carbon acquisition to cope with C limitation.

Improvements in soil moisture and physical properties were important drivers of enzyme activity changes after grazing exclusion. In this study, grazing exclusion significantly increased soil water content (SWC) and decreased bulk density (BD) across the three grasslands (Table 1). Higher soil moisture can accelerate the secretion of extracellular enzymes (German et al., 2012; Stone et al., 2012; He et al., 2025), and lower BD favors water and air infiltration, improving the microhabitat and thus enhancing enzyme activity (Zhang et al., 2018). However, in TS, although SWC and BD improved similarly, enzyme activities decreased, indicating that improvements in moisture and physical properties are not sufficient conditions for increasing enzyme activities; substrate quality and microbial demand must also match.

The accumulation of available resources, such as DOC and DON, after grazing exclusion is an important basis for changes in enzyme activities. Vegetation restoration promotes the accumulation of nutrients and microbial biomass through root exudates and litter inputs (Guo et al., 2019; Li et al., 2020; Xiao et al., 2021). In our study, DOC, DON, and DOP all increased significantly in all grasslands, providing abundant substrates for enzyme synthesis. Nevertheless, enzyme activities increased comprehensively only in TD, decreased in TS, and increased only partially in MM. This indicates that enzyme activities are controlled not only by the total amount of resources but also by the stoichiometric matching of resources. Appendix Supplementary Figure A2 shows that the BG/(LAP+NAG) ratio increased significantly in both TD and TS (by 70.86 and 19.82%, respectively), indicating that grazing exclusion led to a relative increase in microbial investment in C-acquiring enzymes, suggesting enhanced C demand. In TS, although the absolute activity of BG decreased, its ratio to N-acquiring enzymes increased, which may reflect a metabolic trade-off by microorganisms under changing total resource availability.

In summary, the regulation of enzyme activities by grazing exclusion is the result of the combined effects of plant inputs, soil moisture, nutrient availability, and microbial metabolic strategies, and these effects vary markedly among grassland types. In TD, enzyme activities increased comprehensively despite reduced plant biomass, suggesting that grazing exclusion may stimulate microbial activity by increasing litter input and improving soil resource availability. In TS, although plant and soil resources increased, enzyme activities declined, possibly due to intensified plant-microbe competition or an imbalance in microbial C, N, and P demands. In MM, only C-acquiring enzyme activity increased, likely reflecting a relatively prominent C limitation. These results emphasize that grassland-type specific ecological backgrounds must be considered when evaluating the effects of grazing exclusion.

CUE is a key parameter regulating microbial resource allocation and carbon cycling (Manzoni, 2017). In this study, grazing exclusion significantly reduced CUE only in MM (by 15.92%, *P* < 0.01), while no significant changes were observed in TD or TS (Figure 5A). This grassland-type-specific response indicates that the effect of grazing exclusion on microbial carbon metabolism efficiency is not uniform but depends on the background conditions of each grassland ecosystem.

A decline in CUE is often associated with aggravated microbial carbon limitation or decreased substrate quality. Studies have shown that when microorganisms experience nutrient-limited conditions, CUE tends to decrease because microbes allocate more carbon to enzyme production and respiration rather than to biomass synthesis (Mehnaz et al., 2019; Mganga et al., 2022). Moreover, substrate quality is an important factor regulating CUE: simple substrates (e.g., sugars and amino acids) are easily assimilated and result in higher CUE, whereas complex macromolecules (e.g., lignin and cellulose) require multi-step depolymerization, increasing carbon expenditure and thereby reducing CUE (Hu et al., 2022; Gunina and Kuzyakov, 2022; Islam et al., 2023). In the present study, CUE decreased in MM after grazing exclusion, where only C-acquiring enzyme activity increased significantly while N- and P-acquiring enzymes showed no significant change (Figure 3), and vector length (C limitation) increased (Figure 4A). These results indicate that microorganisms in MM may face more severe carbon limitation or decreased substrate quality, leading to reduced metabolic efficiency. In contrast, in TD, enzyme activities increased across the board but CUE did not change significantly, suggesting that microorganisms in this grassland may maintain stable CUE through adjustments in metabolic strategies. In TS, although enzyme activities decreased, CUE also remained unchanged, possibly implying that microorganisms balance carbon metabolism by reducing enzyme investment.

Environmental stress is another important factor influencing CUE. In arid regions, limited soil water availability can maintain relatively high CUE by suppressing microbial metabolic rates (Cui et al., 2024). In this study, TD belongs to an arid region, and although soil water content increased significantly after grazing exclusion, CUE did not increase. This may be due to the limited alleviation of carbon limitation or to microbial adaptation to the arid environment. In contrast, MM belongs to a humid alpine region, where C limitation increased after grazing exclusion, and CUE decreased, suggesting that the inhibitory effect of C limitation on CUE outweighed any potential positive effect of improved soil moisture.

In addition, the quality of plant inputs has a strong influence on CUE. Adequate and high-quality plant inputs (organic matter readily utilizable by microorganisms) can promote rapid microbial growth and turnover, thereby increasing CUE (Craig et al., 2022; Duan et al., 2023). In this study, both aboveground and belowground biomass increased significantly in MM after grazing exclusion, yet CUE decreased, indicating that increased plant carbon input did not alleviate microbial carbon limitation; instead, it might have aggravated microbial metabolic pressure due to changes in litter quality (e.g., increased C:N ratio) or nutrient imbalance. In contrast, in the desert grassland (TD), although plant biomass decreased, CUE remained relatively high, possibly reflecting a conservative microbial metabolic strategy.

In summary, the effect of grazing exclusion on CUE is grassland-type specific: it decreased significantly only in MM, while no significant change was observed in TD or TS. Combining the results of enzyme activities and vector characteristics, the decrease in CUE in MM is closely related to the pronounced increase in carbon limitation (increased vector length) and the lack of response of N- and P-acquiring enzymes. This indicates that the intensification of carbon limitation exceeded the metabolic regulatory capacity of the microorganisms, forcing them to allocate more carbon to respiration rather than growth, thereby reducing CUE. In contrast, in TD and TS, although carbon limitation also increased to some extent, microorganisms effectively buffered the negative impact of carbon limitation on CUE by adjusting enzyme activities (comprehensive increase in TD, relative increase in C-acquiring enzyme investment in TS) or altering resource allocation strategies. Therefore, the change in microbial CUE after grazing exclusion depends not only on the absolute intensity of carbon limitation but also on the balance between microbial metabolic plasticity and environmental stress.

### 4.2 Responses of microbial nutrient limitations (vector characteristics) to grazing exclusion

In this study, vector length increased in all three grassland types after grazing exclusion, reaching highly significant levels in TS and MM, with increments of 1.83 and 8.71%, respectively, indicating that grazing exclusion aggravated soil microbial carbon (C) limitation. Meanwhile, vector angles remained below 45° in all treatments, demonstrating that microorganisms were consistently nitrogen (N)-limited. After grazing exclusion, vector angles increased significantly (by 55.74% in TD, 8.08% in TS, and 11.37% in MM), implying a gradual alleviation of N limitation (Figure 4A). The pattern of enhanced C limitation accompanied by weakened N limitation reveals the differential regulation of microbial nutrient limitation by grazing exclusion.

The enhancement of C limitation was closely related to changes in soil C pools after grazing exclusion. Previous studies have shown that soil total nutrient stoichiometry, especially the soil C:N ratio, strongly influences microbial nutrient limitation (Zhong et al., 2020), and that unbalanced soil elemental stoichiometry can cause shifts in microbial metabolic limitation (Deng et al., 2019; Qiu et al., 2020; Cui et al., 2021; Xiao et al., 2021). In the present study, grazing exclusion significantly increased soil organic carbon (SOC), total nitrogen (TN), total phosphorus (TP), and dissolved organic nutrients (DOC, DON, and DOP) in all three grasslands, yet C limitation was intensified. This paradox may be attributed to the rate of increase in microbial C demand outpacing that of C supply. Importantly, increases in SOC and DOC do not necessarily indicate proportional increases in readily available C substrates for microbial growth, because the accessibility and biochemical quality of organic C regulate microbial C acquisition. During grazing exclusion, accumulated organic matter may be associated with a greater proportion of relatively recalcitrant compounds, which can increase microbial investment in C-acquiring enzymes despite increased soil C pools. Therefore, microbial C limitation is determined not simply by the absolute amount of soil C pools, but by the balance between available C supply, microbial C demand, and elemental stoichiometric constraints. As reported in the literature, variation in soil nutrient stoichiometry is a primary driver of changes in microbial metabolism (Brown et al., 2022; Hao et al., 2023), and shifts in microbial C:N:P ratios are closely linked to changes in soil microbial communities and resource availability (Jiao et al., 2019). After grazing exclusion, microbial biomass (MBC, MBN, and MBP) increased significantly (Figure 2), and such an increase inevitably leads to a higher C demand. When the increased C input is insufficient to meet the energy demands of the enlarged microbial population, C limitation is aggravated. Moreover, although microorganisms adaptively adjust their metabolic strategies to acquire limited nutrients (Ju et al., 2024; Zhou et al., 2024), the persistent intensification of C limitation suggests that, on the timescale of this study (12 years of grazing exclusion), microbial metabolic adjustments could not fully compensate for the C supply-demand gap.

The intensification of C limitation may also be related to changes in litter quality. After grazing exclusion, plant community composition and litter quality changed. In TD, although both aboveground and belowground biomass decreased after grazing exclusion, soil nutrients and microbial biomass increased significantly, implying that the quality (rather than the quantity) of litter input may have changed. Some studies have indicated that an increase in the proportion of recalcitrant components (e.g., lignin) in plant litter reduces C availability, thereby exacerbating C limitation. In addition, the increase in vector length may be indirectly related to phosphorus (P) limitation. According to nutrient stoichiometry theory, the metabolism of one nutrient by microorganisms can also be limited by another nutrient (Yang et al., 2020). To balance nutrient stoichiometry (i.e., maintain homeostasis), microorganisms may need to secrete more C-acquiring enzymes to meet P demand (Cui et al., 2020). Although vector angles remained below 45° (indicating primary N limitation) in all grasslands, the fact that vector angles moved closer to 45° after grazing exclusion suggests a relative increase in the importance of P limitation as N limitation weakened. Such a stoichiometric imbalance may indirectly drive microorganisms to increase investment in C-acquiring enzymes, thus manifesting as an apparent enhancement of C limitation.

In contrast to the enhancement of carbon limitation, the vector angles of all grasslands increased significantly after grazing exclusion, indicating an alleviation of nitrogen limitation. This result differs from the findings of most grassland restoration studies, in which nitrogen limitation typically intensifies with restoration time; the discrepancy may be related to the specific grassland types and the duration of grazing exclusion in this region. In TS and MM, aboveground and belowground biomass increased significantly after grazing exclusion, which would be expected to increase plant N uptake and thus aggravate N limitation. However, the actual result was a weakening of N limitation. This may be because the increase in soil N supply capacity after grazing exclusion exceeded the increase in plant N uptake. In this study, both TN and dissolved organic N (DON) increased significantly after grazing exclusion, providing a more abundant N source for microorganisms. Moreover, studies have shown that microorganisms can cope with nutrient limitation by optimizing resource allocation (Qin et al., 2024; Shen et al., 2023), and microbial biomass stoichiometry regulates nutrient allocation to sustain essential functions (Li et al., 2019). Therefore, after grazing exclusion, microorganisms may have adjusted their C:N:P ratios to utilize available N more efficiently, thereby alleviating N limitation.

The magnitude of changes in vector characteristics varied among the three grassland types. The vector angle increased most strongly in TD (55.74%), whereas its carbon limitation did not increase significantly. In contrast, carbon limitation increased significantly in TS and MM, while the increases in vector angle were relatively smaller (8.08 and 11.37%, respectively). These differences may be attributed to the specific responses of plants and soils to grazing exclusion in each grassland. In TD, both aboveground and belowground biomass decreased after grazing exclusion, yet soil water content, nutrients, and microbial biomass increased significantly. This suggests that the lack of a significant increase in carbon limitation may be due to the partial offsetting of the negative effect of reduced carbon input by improvements in water and nutrient availability; the marked alleviation of N limitation benefited from the substantial increase in soil N availability. In TS, although plant biomass and soil resources both increased significantly, carbon limitation became significantly stronger, implying a decline in carbon quality or an excessively rapid increase in microbial C demand. Nitrogen limitation decreased only slightly, indicating that the increase in N supply only partially alleviated the pre-existing N limitation. In MM, carbon limitation increased significantly while N limitation decreased slightly, a pattern similar to that in TS, but CUE decreased significantly in MM (Figure 5A), suggesting that microorganisms in MM may face stronger metabolic pressure.

Notably, vector length and vector angle showed a positive correlation (Figure 4E). Although the correlation is numerically positive, its ecological implication is inverse: increased carbon limitation is accompanied by decreased nitrogen limitation. This relationship indicates a metabolic trade-off between microbial acquisition of carbon and nitrogen. When carbon limitation intensifies, microorganisms may allocate more energy to carbon acquisition and relatively less to nitrogen acquisition; conversely, when nitrogen limitation is alleviated, microorganisms can allocate more resources to carbon metabolism. This trade-off reflects the metabolic plasticity of microorganisms under resource-limited conditions and explains why enhanced carbon limitation and weakened nitrogen limitation occur simultaneously after grazing exclusion.

In summary, grazing exclusion differentially regulates microbial carbon and nitrogen limitations by altering the balance of soil carbon and nitrogen supply. The enhancement of carbon limitation and the alleviation of nitrogen limitation after grazing exclusion reflect the dual effects of soil C supply-demand imbalance and increased N availability. The differences in responses among contrasting grassland ecosystems highlight the importance of environmental background conditions in regulating the effects of grazing exclusion.

### 4.3 Differential pathways associated with microbial C/N limitations and CUE under grazing exclusion: implications for grassland restoration

PLS-SEM revealed distinct patterns of direct and indirect associations among grazing exclusion, plant and soil properties, microbial nutrient limitations (C and N limitation), and carbon use efficiency (CUE) across the three grassland types (Figure 7). The temperate desert, temperate steppe, and mountain meadow exhibited different path structures, indicating that the associations between grazing exclusion and microbial metabolism were strongly grassland-type specific and varied with the background climate, plant communities, and soil properties of each grassland.

In TD, grazing exclusion was negatively associated with plant belowground biomass (BGB), which in turn showed a negative association with the N limitation pattern (path coefficient = −0.88, *P* < 0.001). At the same time, grazing exclusion was positively associated with soil properties (SWC, TP, and SOC/TP), which were associated with dissolved organic nutrients (DOC/DOP) and subsequently with C limitation (vector length) and CUE (Figure 7A). This path structure reflects the uniqueness of TD: after grazing exclusion, both aboveground and belowground biomass decreased, yet soil water content, nutrients, and microbial biomass increased significantly. Consequently, the effect of grazing exclusion was transmitted mainly through an indirect pathway of “soil properties → dissolved nutrients → microorganisms”, and the negative association involving plant BGB may be consistent with reduced plant–microbe competition or changes in root-derived C inputs after grazing exclusion. Previous studies have shown that soil dissolved nutrients and microbial biomass have stronger effects on enzyme activities and ecoenzymatic stoichiometry than plants or other soil properties, because microbial nutrient demand is determined by both microbial biomass and environmental nutrient availability (Cui et al., 2018). In TD, variation in C limitation and CUE was most strongly associated with soil properties and dissolved nutrients, consistent with the dominance of soil resources in microbial metabolism under resource-limited arid conditions.

In TS, the pathway structure was characterized by positive associations between grazing exclusion and soil properties (SWC, pH, and SOC), which were subsequently associated with the N limitation pattern (path coefficient = 0.31, *P* < 0.05) and indirectly associated with C limitation through microbial biomass (MBC, MBN; path coefficient = 0.86, *P* < 0.01; Figure 7C). After grazing exclusion in TS, both aboveground and belowground biomass increased substantially, and soil water content, organic carbon, and pH improved markedly, all of which were positively associated with microbial activity. Unlike TD, the pathway in TS was shorter and more direct, reflecting a positive plant-soil-microbe feedback. Studies have demonstrated that soil properties have the strongest effect on microbial metabolic limitation, as soil provides nutrients and water to sustain microbial growth (Liu et al., 2013; Deng et al., 2019), and plant composition and litter quality indirectly affect microbial nutrient limitation by influencing microbial composition and soil nutrient supply (Cui et al., 2019). The pathway involving microbial biomass suggests that variation in microbial biomass was strongly associated with C limitation in TS, consistent with the possibility that the increased microbial biomass was accompanied by greater microbial C demand after grazing exclusion.

In MM, the pathway structure was the most complex. Grazing exclusion was positively associated with soil properties (SWC, SOC, SOC/TN, and SOC/TP), which were subsequently associated with the N limitation pattern (path coefficient = 0.80, *P* < 0.05). Grazing exclusion was also associated with dissolved organic nutrients (DOC, DON, DOP, and DON/DOP), which were linked to variation in microbial biomass. From microbial biomass, the pathway bifurcated into two branches: one was positively associated with C limitation (path coefficient = 0.88, *P* < 0.001), whereas the other was negatively associated with CUE (path coefficient = −0.83, *P* < 0.01; Figure 7E). This long cascade pathway indicates that, in MM, the associations between grazing exclusion and C limitation and CUE were characterized by the chain “soil properties → dissolved nutrients → microbial biomass”, and the increase in microbial biomass was accompanied by stronger C limitation and lower CUE. The combined effects of plant, soil, and microbial processes jointly influence microbial metabolism and its limitations (Deng et al., 2019). The negative association between microbial biomass and CUE in MM contrasts sharply with the patterns observed in the other two grasslands. This pattern may be related to the stronger C limitation observed after grazing exclusion in the MM. The substantial increase in microbial biomass may have been accompanied by greater microbial C demand, whereas the associated changes in dissolved nutrient supply may not have fully matched this demand. Under such conditions, a greater proportion of available C may have been allocated to maintenance and metabolic processes rather than biomass production, potentially contributing to the observed decline in CUE (Li S. Y. et al., 2024).

Total effects analysis (Figures 7B, D, F) further revealed the relative importance of key factors in the different grassland types. In TD, grazing exclusion, plant traits (BGB), and microbial biomass (MBN, MBP, and MBC/MBP) showed the strongest associations with N limitation, whereas soil properties (SWC, TP, and SOC/TP) and dissolved nutrients (DOC/DOP) were most strongly associated with C limitation and CUE. In TS, grazing exclusion, soil properties, and microbial biomass showed strong associations with C and N limitations, while dissolved nutrients and microbial biomass were most strongly associated with CUE. In MM, grazing exclusion and soil properties showed the strongest associations with N limitation, whereas soil properties and microbial biomass showed the strongest associations with C limitation and CUE. The PLS-SEM results are consistent with the view that total soil nutrient content is closely associated with soil nutrient stoichiometry, which in turn is associated with microbial metabolic limitations (Xu W. X. et al., 2024). In terms of total effects, soil nutrient were negatively associated with C limitation but positively associated with N limitation (ibid.), a pattern observed in all three grasslands of this study. The differences in total effect factors among grassland types reflect the profound influence of background conditions. Variations in the relationships between environmental factors and enzyme activities across different study areas are likely attributable to differences in vegetation type, soil properties, and climatic conditions (Yang et al., 2020; Long et al., 2024). Therefore, the observed associations between grazing exclusion and microbial nutrient limitation appear to reflect the combined variation in plants, soil, and microorganisms, and the relative importance of each factor varies with grassland type.

The differences in regulation pathways among the three grassland types can be explained by their ecosystem backgrounds. TD is a temperate desert with extremely limited water and nutrients. After grazing exclusion, plant biomass decreased, but soil water and nutrients improved; thus, microbial metabolism appeared to be more closely associated with soil resource availability than with plant inputs. TS is a semi-arid typical steppe, where plant and soil resources improved synchronously after grazing exclusion, forming a positive plant-soil-microbe feedback with a short and direct pathway. MM is a mountain meadow; although plant biomass and soil resources both increased significantly after grazing exclusion, C limitation intensified, and CUE decreased, which may be related to reduced litter quality (e.g., an increase in the proportion of recalcitrant C such as lignin) and an elevated metabolic cost for microorganisms. Studies have shown that vegetation restoration increases microbial biomass and alters soil microbial structure, thereby potentially affecting microbial C limitation (Deng et al., 2019). In MM, the large increase in microbial biomass was accompanied by greater potential C demand, while the cascade of associations involving dissolved nutrients may not have fully matched this demand, which may help explain the observed enhancement of C limitation and reduction in CUE.

From the perspective of grassland restoration management, the effect of grazing exclusion is not “one-size-fits-all”. In TD, grazing exclusion was associated with improved microbial function alongside enhanced soil water and nutrient status; despite the decrease in plant biomass, improved soil resource availability may have partially offset the reduced plant input, suggesting that grazing exclusion can be an effective restoration measure in TD. In TS, grazing exclusion was associated with enhanced microbial metabolism alongside positive plant-soil-microbe relationships, but C limitation still increased, highlighting the need to pay attention to changes in carbon quality (e.g., litter C:N ratio). In MM, although grazing exclusion was associated with increased soil resource availability, C limitation intensified and CUE decreased, suggesting that complete grazing exclusion in MM may be associated with a potential “metabolic cost” and may require supplementary measures (e.g., moderate disturbance or nutrient regulation) to alleviate C limitation. Microbial nutrient limitation is the result of the combined interaction of plants, soil, and microorganisms (Mu et al., 2025). It should be noted that the PLS-SEM results represent statistical associations consistent with the hypothesized ecological pathways rather than causal effects. Because the present study was based on a cross-sectional comparison of grazing exclusion and freely grazed plots, the observed path relationships cannot establish temporal or causal relationships among grazing exclusion, plant traits, soil properties, microbial biomass, microbial nutrient limitation, and CUE. Therefore, the pathways identified here should be interpreted as hypothesized association pathways rather than definitive causal mechanisms. Long-term monitoring, controlled manipulation experiments, and repeated measurements across restoration chronosequences are needed to further verify these proposed pathways.

Nevertheless, we acknowledge that the estimation of microbial CUE based on the biogeochemical equilibrium model involves several assumptions. Applying identical parameter values across contrasting grassland ecosystems represents a simplification of the model. Differences in climate, soil properties, and microbial communities among TD, TS, and MM may influence the actual physiological constraints on microbial carbon use efficiency. Therefore, the estimated CUE values should be interpreted as relative indicators reflecting microbial carbon allocation responses to grazing exclusion rather than absolute measurements of microbial metabolic efficiency. Further refinement of CUE estimation through ecosystem-specific parameterization or direct measurements of microbial carbon allocation would help improve the accuracy of microbial CUE assessments.

In addition, although our comparison among three grassland ecosystems provides insights into ecosystem-specific responses to grazing exclusion, each ecosystem type was represented by a single geographical region; therefore, the observed differences may reflect both ecosystem characteristics and site-specific climatic and edaphic conditions. Multi-site studies across broader environmental gradients are needed to further disentangle ecosystem-level effects from site-specific environmental variations. Therefore, grassland restoration strategies should be tailored to local conditions, taking full account of ecosystem-specific environmental contexts. Vector length and vector angle can serve as sensitive bioindicators for assessing grassland quality. Future studies should further investigate the functional succession of microbial communities under long-term grazing exclusion chronosequences and integrate multi-omics techniques to resolve the molecular mechanisms of microbial metabolic regulation, thereby deepening our understanding of soil carbon cycling during grassland restoration.

## 5 Conclusions

In this study, we systematically investigated the effects of 12-year grazing exclusion vs. free grazing on soil extracellular enzyme activities, microbial nutrient limitation (vector characteristics), and CUE across three typical grassland types in Xinjiang: temperate desert (TD), temperate steppe (TS), and mountain meadow (MM). Our results demonstrate that the effects of grazing exclusion on enzyme activities are grassland-type specific: all three enzyme activities increased in TD; C- and N-acquiring enzymes decreased in TS; only C-acquiring enzyme increased in MM. Grazing exclusion generally aggravated microbial C limitation (increased vector length) while alleviated N limitation (vector angle approaching 45°), yet all vector angles remained below 45°, indicating persistent N limitation. CUE decreased significantly only in MM, which was closely linked to aggravated C limitation and the lack of response of N- and P-acquiring enzymes. Structural equation modeling revealed distinct regulation pathways among the three grasslands: an indirect soil-to-dissolved-nutrient-to-microbial biomass pathway dominated in TD; a direct soil-to-microbial biomass pathway dominated in TS; and a long cascade pathway that positively influenced C limitation but negatively influenced CUE in MM. Our findings demonstrate that the regulation of grassland microbial metabolism by grazing exclusion is strongly grassland-type dependent. Grazing exclusion was associated with improvements in microbial metabolic conditions in TD and TS, whereas the increased C limitation and decreased CUE observed in MM suggest a potential metabolic constraint under the 12-year restoration period. However, these ecosystem-specific responses are based on a single sampling time point and should not be interpreted as definitive long-term restoration trajectories. Therefore, grazing exclusion management should be evaluated using multiple indicators, including vegetation productivity, biodiversity, soil health, and microbial metabolic characteristics. Long-term monitoring, including restoration periods beyond 20 years and chronosequence studies, is needed to determine whether these patterns persist or change over time. Vector length and vector angle can serve as sensitive bioindicators of microbial nutrient limitation during grassland restoration. This study provides insights into the potential mechanisms underlying the paradox of increased soil carbon pools but intensified microbial C limitation under grazing exclusion, and provides a basis for developing site-specific restoration strategies across different grassland types.

## Data Availability

The original contributions presented in the study are included in the article/Supplementary material, further inquiries can be directed to the corresponding author.
