# Supplementary material for: Differential responses of soil microbial metabolic limitation and carbon use efficiency to long-term grazing exclusion across three grassland types in Xinjiang
Source: Front Microbiol. 2026 Sep 11;17:1905160. doi: 10.3389/fmicb.2026.1905160 (PMC13612500; doi:10.3389/fmicb.2026.1905160)
Supplement: Supplementary file 1 [file Supplementary_file_1.docx]

**Appendix Supporting information**

**1.1 Effects of grazing exclusion on stoichiometric ratios of soil dissolved organic nutrients and microbial biomass**

Grazing exclusion significantly altered the stoichiometric ratios of soil dissolved organic nutrients (DOC/DON, DOC/DOP, DON/DOP) across the three grassland types (Fig. A1). After grazing exclusion, only in the mountain meadow (MM) did DOC/DON decrease significantly by 57.38%, while DON/DOP increased significantly by 107.77% (*P* < 0.05). In the other grassland types, most of the dissolved nutrient ratios decreased but not significantly (*P* > 0.05; Fig. A1a-c).

Similarly, the stoichiometric ratios of soil microbial biomass (MBC/MBN, MBC/MBP, MBN/MBP) showed analogous patterns. In MM, MBC/MBN decreased significantly by 57.43%, whereas MBN/MBP increased significantly by 106.81% (*P* < 0.05). In the other grasslands, most microbial biomass ratios also decreased but without statistical significance (*P* > 0.05; Fig. A1a-c).

Two-way ANOVA revealed that, for main effects, grazing exclusion had highly significant effects on DOC/DON, MBC/MBN, and MBN/MBP (*P* < 0.01), while grassland type had highly significant effects on DOC/DON, DOC/DOP, DON/DOP, MBC/MBN, and MBN/MBP (*P* < 0.01). Moreover, the interaction between grazing exclusion and grassland type exerted highly significant effects on DOC/DON, DON/DOP, MBC/MBN, and MBN/MBP (*P* < 0.001; Fig. A1a-c).

Figure A1. Stoichiometric ratios of soil dissolved organic nutrients and microbial biomass under grazing exclusion across three grassland types. (a) DOC/DON and MBC/MBN; (b) DOC/DOP and MBC/MBP; and (c) DON/DOP and MBN/MBP. All data points and error bars represent mean ± standard error. “*”, “**”, and “***” indicate significant differences at *P* < 0.05, *P* < 0.01, and *P* < 0.001, respectively, between grazing treatments within the same grassland type, as determined by independent-sample t-tests, whereas “ns” indicates no significant difference (*P* > 0.05). Capital letters indicate significant differences among grassland types under freely grazing conditions, whereas lowercase letters indicate significant differences among grassland types under grazing exclusion conditions (*P* < 0.05), as determined by one-way ANOVA followed by Duncan’s multiple range test. Gray boxes above the histograms show the results of two-way ANOVA for the displayed metric. Box plots show the combined effects of grazing exclusion and grassland type across all three grassland types, as determined by one-way ANOVA followed by Duncan’s multiple range test. TD, temperate desert; TS, temperate steppe; MM, mountain meadow; GE, grazing exclusion plots; FG, freely grazing plots.

**1.2 Effects of grazing exclusion on stoichiometric ratios of soil enzyme activities**

As shown in Fig. A2, grazing exclusion significantly altered the ecoenzymatic stoichiometric ratios. In TD and TS, only BG/(LAP+NAG) increased significantly, by 70.86% and 19.82%, respectively (*P* < 0.05; Fig. A2a). In MM, all three ratios increased to varying degrees, with BG/(LAP+NAG) and BG/AP increasing significantly by 38.01% and 47.01% (*P* < 0.05; Fig. A2a-b).

Two-way ANOVA revealed that, for main effects, both grazing exclusion and grassland type had significant effects on BG/(LAP+NAG), BG/AP, and (LAP+NAG)/AP (*P* < 0.05). Moreover, the interaction between grazing exclusion and grassland type exerted highly significant effects on all three ratios (*P* < 0.01; Fig. A2a-c).

Figure A2. Stoichiometric ratios of soil enzyme activities under grazing exclusion across three grassland types. (a) BG/(LAP+NAG); (b) BG/AP; and (c) (LAP+NAG)/AP. All data points and error bars represent mean ± standard error. “*”, “**”, and “***” indicate significant differences at *P* < 0.05, *P* < 0.01, and *P* < 0.001, respectively, between grazing treatments within the same grassland type, as determined by independent-sample t-tests, whereas “ns” indicates no significant difference (*P* > 0.05). Capital letters indicate significant differences among grassland types under freely grazing conditions, whereas lowercase letters indicate significant differences among grassland types under grazing exclusion conditions (*P* < 0.05), as determined by one-way ANOVA followed by Duncan’s multiple range test. Gray boxes above the histograms show the results of two-way ANOVA for the displayed metric. Box plots show the combined effects of grazing exclusion and grassland type across all three grassland types, as determined by one-way ANOVA followed by Duncan’s multiple range test. TD, temperate desert; TS, temperate steppe; MM, mountain meadow; GE, grazing exclusion plots; FG, freely grazing plots.

**1.3 Correlations of vector characteristics and soil CUE with environmental factors under grazing exclusion**

Using Spearman correlation analysis, we evaluated the associations between plant traits, soil physicochemical properties, dissolved organic nutrients, microbial biomass, and vector characteristics as well as soil CUE. The results are shown in Fig. A3 and have been presented in the main text (Section 3.5).

Figure A3. Spearman correlation heatmaps between environmental factors (plant traits, soil physicochemical properties, dissolved organic nutrients, and microbial biomass) and vector characteristics (length and angle) and CUE across three grassland types. Color intensity represents the strength and direction of Spearman correlations, with orange indicating positive correlations and blue indicating negative correlations. “*”, “**”, and “***” indicate significant correlations at *P* < 0.05, *P* < 0.01, and *P* < 0.001, respectively. TD, temperate desert; TS, temperate steppe; MM, mountain meadow; CUE, microbial carbon use efficiency.

**1.3 Environmental controls on vector characteristics and soil CUE revealed by PLS-PM under grazing exclusion**

Table A1. Reliability and convergent validity assessment of latent constructs.

| **Construct** | **TD model** |  |  | **TS model** |  |  | **MM model** |  |  |
| --- | --- | --- | --- | --- | --- | --- | --- | --- | --- |
|  | **R²** | **AVE** | **CR** | **R²** | **AVE** | **CR** | **R²** | **AVE** | **CR** |
| Plant characteristics | 0.760 | 1.000 | 1.000 | 0.945 | 0.903 | 0.949 | 0.919 | 0.769 | 0.908 |
| Soil properties | 0.770 | 0.719 | 0.573 | 0.950 | 0.828 | 0.955 | 0.973 | 0.924 | 0.980 |
| Dissolved organic nutrients | 0.638 | 1.000 | 1.000 | 0.899 | 0.928 | 0.963 | 0.934 | 0.871 | 0.964 |
| Microbial biomass | 0.932 | 0.824 | 0.691 | 0.942 | 0.938 | 0.967 | 0.982 | 0.952 | 0.952 |
| Carbon limitation | 0.316 | 1.000 | 1.000 | 0.740 | 1.000 | 1.000 | 0.781 | 1.000 | 1.000 |
| Nitrogen limitation | 0.961 | 1.000 | 1.000 | 0.965 | 1.000 | 1.000 | 0.861 | 1.000 | 1.000 |
| CUE | 0.901 | 1.000 | 1.000 | 0.026 | 1.000 | 1.000 | 0.683 | 1.000 | 1.000 |

Table A2. Outer loadings of indicators for latent constructs in the PLS-PM model

| **Latent construct** | **TD model** |  | **TS model** |  | **MM model** |  |
| --- | --- | --- | --- | --- | --- | --- |
|  | **Indicator** | **Outer loading** | **Indicator** | **Outer loading** | **Indicator** | **Outer loading** |
| Plant characteristics | BGB | 1.000 | AGB | 0.945 | AGB | 0.873 |
|  |  |  | BGB | 0.955 | BGB | 0.977 |
|  |  |  |  |  | Shannon-Wiener index | 0.769 |
| Soil properties | SWC | 0.852 | SWC | 0.929 | SWC | 0.971 |
|  | TP | 0.971 | pH | -0.875 | SOC | 0.979 |
|  | SOC/TP | -0.698 | SOC | 0.927 | SOC/TN | 0.977 |
|  |  |  |  |  | SOC/TP | 0.916 |
| Dissolved organic nutrients | DOC/DOP | 1.000 | DOC | 0.968 | DOC | 0.971 |
|  |  |  | DON | 0.958 | DON | 0.993 |
|  |  |  |  |  | DOP | 0.833 |
|  |  |  |  |  | DON/DOP | 0.928 |
| Microbial biomass | MBN | 0.970 | MBC | 0.963 | MBC | 0.950 |
|  | MBP | 0.963 | MBN | 0.973 | MBN | 0.999 |
|  | MBC/MBP | -0.779 |  |  | MBC/MBN | -0.979 |
|  |  |  |  |  | MBN/MBP | 0.975 |
| Carbon limitation | length | 1.000 | length | 1.000 | Vector length | 1.000 |
| Nitrogen limitation | angle | 1.000 | angle | 1.000 | Vector angle | 1.000 |
| CUE | CUE | 1.000 | CUE | 1.000 | CUE | 1.000 |
